# Whole Exome Sequencing Identifies *TSC1/TSC2* Biallelic Loss as the Primary and Sufficient Driver Event for Renal Angiomyolipoma Development

**DOI:** 10.1371/journal.pgen.1006242

**Published:** 2016-08-05

**Authors:** Krinio Giannikou, Izabela A. Malinowska, Trevor J. Pugh, Rachel Yan, Yuen-Yi Tseng, Coyin Oh, Jaegil Kim, Magdalena E. Tyburczy, Yvonne Chekaluk, Yang Liu, Nicola Alesi, Geraldine A. Finlay, Chin-Lee Wu, Sabina Signoretti, Matthew Meyerson, Gad Getz, Jesse S. Boehm, Elizabeth P. Henske, David J. Kwiatkowski

**Affiliations:** 1 Division of Pulmonary and Critical Care Medicine and of Genetics, Brigham and Women’s Hospital, Harvard Medical School, Boston, Massachusetts, United States of America; 2 Broad Institute of Harvard and MIT, Cambridge, Massachusetts, United States of America; 3 Tufts New England Medical Center, Boston, Massachusetts, United States of America; 4 Massachusetts General Hospital, Boston, Massachusetts, United States of America; Stanford University School of Medicine, UNITED STATES

## Abstract

Renal angiomyolipoma is a kidney tumor in the perivascular epithelioid (PEComa) family that is common in patients with Tuberous Sclerosis Complex (TSC) and Lymphangioleiomyomatosis (LAM) but occurs rarely sporadically. Though histologically benign, renal angiomyolipoma can cause life-threatening hemorrhage and kidney failure. Both angiomyolipoma and LAM have mutations in *TSC2* or *TSC1*. However, the frequency and contribution of other somatic events in tumor development is unknown. We performed whole exome sequencing in 32 resected tumor samples (n = 30 angiomyolipoma, n = 2 LAM) from 15 subjects, including three with TSC. Two germline and 22 somatic inactivating mutations in *TSC2* were identified, and one germline *TSC1* mutation. Twenty of 32 (62%) samples showed copy neutral LOH (CN-LOH) in *TSC2* or *TSC1* with at least 8 different LOH regions, and 30 of 32 (94%) had biallelic loss of either *TSC2* or *TSC1*. Whole exome sequencing identified a median of 4 somatic non-synonymous coding region mutations (other than in *TSC2/TSC1*), a mutation rate lower than nearly all other cancer types. Three genes with mutations were known cancer associated genes (*BAP1*, *ARHGAP35* and *SPEN*), but they were mutated in a single sample each, and were missense variants with uncertain functional effects. Analysis of sixteen angiomyolipomas from a TSC subject showed both second hit point mutations and CN-LOH in *TSC2*, many of which were distinct, indicating that they were of independent clonal origin. However, three tumors had two shared mutations in addition to private somatic mutations, suggesting a branching evolutionary pattern of tumor development following initiating loss of *TSC2*. Our results indicate that *TSC2* and less commonly *TSC1* alterations are the primary essential driver event in angiomyolipoma/LAM, whereas other somatic mutations are rare and likely do not contribute to tumor development.

## Author Summary

We performed comprehensive genome analysis of a kidney tumor called angiomyolipoma. These tumors are known to develop in most individuals who have Tuberous Sclerosis Complex (TSC) and those who have sporadic lymphangioleiomyomatosis (LAM), and are seen rarely in the general population. In these angiomyolipomas, we found consistent involvement of the *TSC2* and *TSC1* genes that are known to cause TSC, but very few (<5 on average) mutations elsewhere in the protein-coding regions. This is in stark contrast to other adult solid tumours that typically harbor hundreds to thousands of such mutations. Our results indicate that genetic alterations in *TSC2/TSC1* are the primary and essential driver genetic events for development and progression of renal angiomyolipoma. Analysis of multiple angiomyolipomas from a single patient showed distinct genetic aberrations in the majority of samples, indicating that most of the tumors had developed independently. Branched clonal evolution was evident from the observation of three tumors that shared 2 mutations in addition to mutations private to each. Our results indicate that therapeutic approaches for treatment of patients with angiomyolipoma should focus on the consequences of *TSC2/TSC1* loss, including but not limited to mTOR activation.

## Introduction

Renal angiomyolipoma is a pathologically benign mesenchymal kidney tumor, characterized by vascular, smooth muscle, and adipocyte elements [1, 2]. Angiomyolipomas are rare in the general population (autopsy prevalence about 1 in 1,000 [3]) but are seen in > 70% adults with Tuberous Sclerosis Complex (TSC (MIM: 191100, 613254 [4]) where they are usually multifocal and bilateral [5]. TSC is a genetic disorder characterized by seizures, tumor development in the brain, heart, kidney, and skin, and a distinctive set of neurodevelopmental syndromes known as TSC-associated neurologic disorder (TAND) [6, 7]. TSC is due to inactivating heterozygous or mosaic mutations in either *TSC1* (~21%) or *TSC2* (~79%) [6]. Lymphangioleiomyomatosis (LAM) is a pathologic condition related to angiomyolipoma in which a similar cellular proliferation occurs in the lung in the form of small (< 5 mm) nodules, causing progressive cystic lung disease that can be fatal [8]. LAM occurs almost exclusively in women, and is seen at much higher frequency in TSC than in the general population, with up to 80% of women with TSC having evidence of cystic lung disease [9]. Both angiomyolipoma and LAM belong to the family of Perivascular Epithelioid Cell tumors (PEComa) [10, 11].

Previous genetic studies have shown that angiomyolipomas arising in TSC patients occur due to biallelic inactivation of either *TSC2* or *TSC1* [1]. The first mutational event (‘hit’) in *TSC2* or *TSC1* is the germline mutation that is the cause of TSC in the individual. The genetic “second hit” event leads to hyperactivation of mTORC1 (mammalian Target Of Rapamycin Complex 1) and contributes to tumor development [12, 13]. Sporadic, non-TSC associated, renal angiomyolipoma and LAM are due nearly exclusively to mutations in *TSC2* [14, 15]. Concurrent analysis of angiomyolipomas and LAM lung samples from women with sporadic LAM but not TSC has demonstrated that these two lesions have identical point mutations in *TSC2*, providing strong evidence that they are clonal and derived from a common cell [16]. However, *TSC2* mutations have not been identified in all cases of LAM, possibly due to technical limitations related to the small amount of tumor tissue available [14]. Angiomyolipoma samples from patients with either sporadic or TSC-associated tumors often display loss of the wild type allele (loss of heterozygosity, LOH) for *TSC2* [17, 18] [19], as well as evidence of activation of mTORC1 [12, 13, 19].

It is unknown whether additional genetic events, beyond loss of *TSC1* or *TSC2*, contribute to angiomyolipoma development, particularly in large tumors requiring surgical resection. We report here the first exome-wide genetic analysis of these tumors.

## Results

### Samples

**Fig 1:**
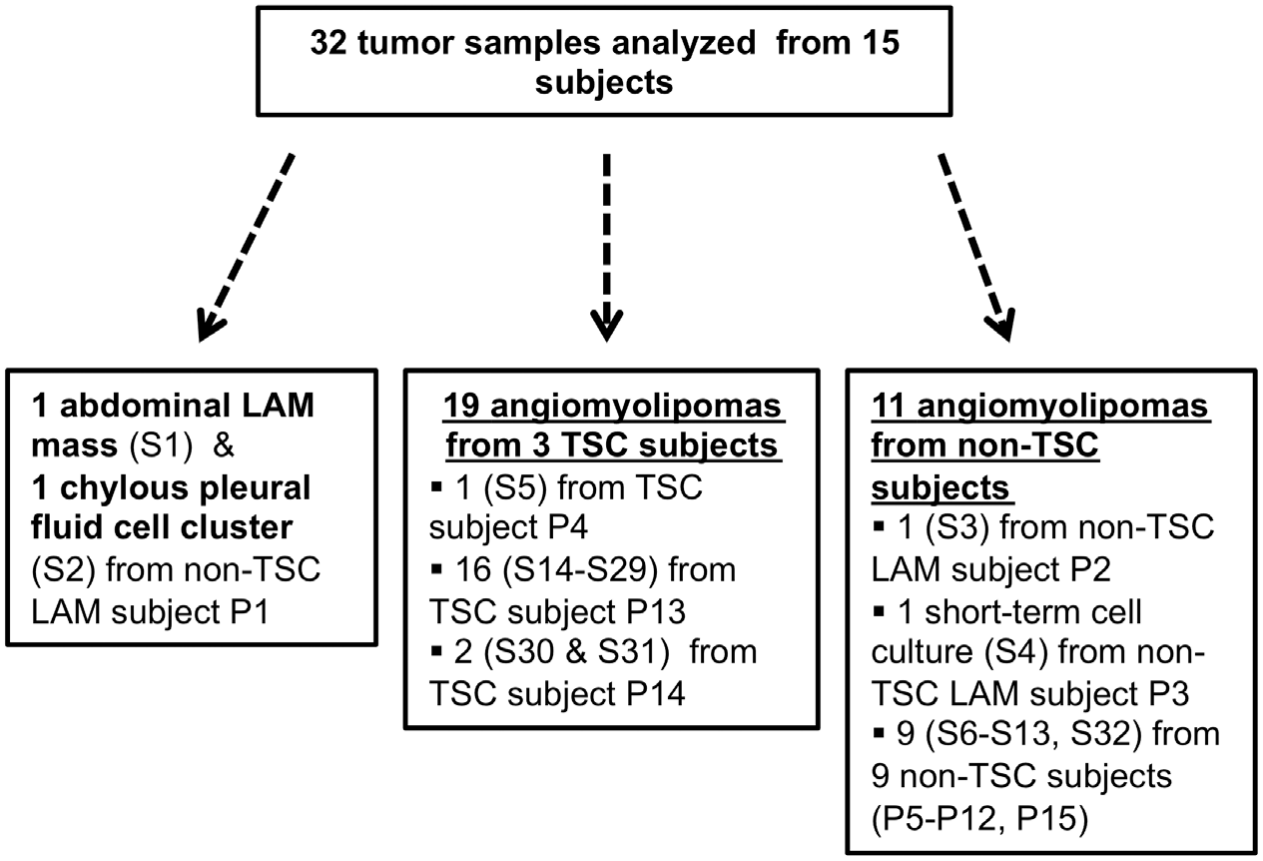
The diagram shows the different kinds of tumor samples analyzed from the different kinds of patients. S#: Sample number, P#: Patient number.

Thirty-two tumor samples (n = 30 renal angiomyolipomas; n = 2 LAM) from 15 patients were examined for somatic mutations by performing whole exome sequencing (Table 1, Fig 1). For one subject with LAM but not TSC (P1), we analyzed both a resected abdominal LAM tumor and a LAM cell cluster isolated from chylous pleural fluid. Nineteen angiomyolipoma samples were available from 3 TSC subjects, including one subject undergoing bilateral kidney removal (P13), from whom 16 macroscopically-distinct renal angiomyolipomas (S14-S29) were available, with 12 tumors from the left kidney and 4 from the right (S1 Fig). Single angiomyolipoma samples were collected from 11 subjects without TSC (P2, P3, P5-P12, P15), of whom two had LAM. All samples were operative samples reflecting the clinical situation that these tumors were relatively large necessitating surgical intervention.

**Table 1:** LIst of patient samples available, and mutation findings in *TSC2* and *TSC1*.

Patient #	Sample #	Source	Variant Type	TSC2 cDNA NM_000548.3	TSC2 aa Uniprot P49815.2	Mutant allele fraction	Mutant alleleread count	Reference allele read count	16p LOH	Other notes*
P1	S1	abdominal LAM	Deletion	c.2319delA	p.L773fs	0.54	49	41	Yes	WGS, RNA-Seq
P1	S2	chylous fluid cell cluster	Deletion	c.2319delA	p.L773fs	0.39	5	8	Yes	
P2	S3	angiomyolipoma	Splice	c.976-15G>A	p.A326_splice	0.81	67	16	Yes	WGS, RNA-Seq, LAM
P3	S4	angiomyolipoma cell line	Deletion	c.2250delC	p.L750fs	0.60	55	36	Yes	LAM
P4	S5	angiomyolipoma	Splice	c.4493+1G>A	p.S1498_splice	0.41	28	41	2-hit	RNA-Seq, TSC
P4	S5	angiomyolipoma	Deletion	c.4765delC	p.P1589fs	0.44	28	36		
P5	S6	angiomyolipoma	Nonsense	c.1195G>T	p.E399*	0.34	25	48	2-hit	RNA-Seq
P5	S6	angiomyolipoma	Deletion	c.2246delG	p.R749fs	0.26	21	61		
P6	S7	angiomyolipoma	Genomic deletion	NA	NA	0.63	NA	NA	NA	RNA-Seq
P7	S8	angiomyolipoma	Splice	c.2742+1G>A	p.K914_splice	0.28	15	38	2-hit	no normal
P7	S8	angiomyolipoma	In-frame deletion	c.5227_5244delCGGCTCCGCCACATCAAG	p.RLRHIK1743del	0.08	18	215		no normal
P8	S9	angiomyolipoma	Nonsense	c.3581G>A	p.W1194*	0.36	56	100	2-hit	no normal
P8	S9	angiomyolipoma	Deletion	c.4565_4566delAT	p.N1522fs	0.25	42	123		no normal
P9	S10	angiomyolipoma	Splice	c.3132-1G>C	p.R1044_splice	0.32	35	73	2-hit	no normal
P9	S10	angiomyolipoma	Nonsense	c.4905C>A	p.C1635*	0.33	15	31		no normal
P10	S11	angiomyolipoma	Nonsense	c.3310C>T	p.Q1104*	0.35	8	15	2-hit	no normal
P10	S11	angiomyolipoma	Splice	c.976-15G>A	p.A326_splice	0.23	44	144		no normal
P11	S12	angiomyolipoma	Nonsense	c.274G>T	p.E92*	0.38	6	10	2-hit	no normal
P11	S12	angiomyolipoma	Nonsense	c.3259G>T	p.E1087*	0.24	38	119		no normal
P12	S13	angiomyolipoma	Nonsense	c.4762C>T	p.Q1588*	0.67	45	22	Yes	no normal
P13	S14	angiomyolipoma	Deletion	c.5135delC	p.A1712fs	0.72	84	32	Yes	TSC
P13	S15	angiomyolipoma	Deletion	c.5135delC	p.A1712fs	0.60	370	248	Yes	TSC
P13	S16	angiomyolipoma	Deletion	c.5135delC	p.A1712fs	0.36	69	121	No	TSC
P13	S17	angiomyolipoma	Deletion	c.5135delC	p.A1712fs	0.74	119	42	Yes	TSC
P13	S18	angiomyolipoma	Deletion	c.5135delC	p.A1712fs	0.75	102	34	Yes	TSC
P13	S19	angiomyolipoma	Deletion	c.5135delC	p.A1712fs	0.53	86	76	Yes	TSC
P13	S20	angiomyolipoma	Deletion	c.5135delC	p.A1712fs	0.64	96	53	Yes	TSC
P13	S21	angiomyolipoma	Deletion	c.5135delC	p.A1712fs	0.64	93	53	Yes	TSC
P13	S22	angiomyolipoma	Deletion	c.5135delC	p.A1712fs	0.50	95	97	Yes	TSC
P13	S23	angiomyolipoma	Deletion	c.5135delC	p.A1712fs	0.52	92	84	Yes	TSC
P13	S24	angiomyolipoma	Deletion	c.5135delC	p.A1712fs	0.70	114	48	Yes	TSC
P13	S25	angiomyolipoma	Deletion	c.5135delC	p.A1712fs	0.71	129	54	Yes	TSC
P13	S26	angiomyolipoma	Deletion	c.5135delC	p.A1712fs	0.43	77	104	2-hit	TSC
P13	S26	angiomyolipoma	Insertion	c.888_889insT	p.F297fs	0.31	155	345		TSC
P13	S27	angiomyolipoma	Deletion	c.5135delC	p.A1712fs	0.35	73	134	2-hit	TSC
P13	S27	angiomyolipoma	Insertion	c.2035-2036insG	p.V679fs	0.12	26	185		TSC
P13	S28	angiomyolipoma	Deletion	c.5135delC	p.A1712fs	0.34	72	137	No	TSC
P13	S29	angiomyolipoma	Deletion	c.5135delC	p.A1712fs	0.74	145	51	Yes	TSC
P14	S30	angiomyolipoma	Nonsense	c.2074C>T	TSC1 p.R692*	0.72	121	46	9q LOH	no normal, TSC
P14	S371	angiomyolipoma	Nonsense	c.2074C>T	TSC1 p.R692*	0.58	83	60	9q LOH	no normal, TSC
P15	S32	angiomyolipoma	Splice	c.2742+1G>A	p.K914_splice	0.50	125	123	Yes	no normal

*Entries in this column indicate: WGS—also subject to whole genome sequencing; RNA-Seq—also subject to RNA-Seq analysis; TSC—diagnosis of TSC; LAM—diagnosis of LAM; no normal—no normal sample available for comparison.

### Mutation and LOH findings in *TSC2* and *TSC1*

**Fig 2:**
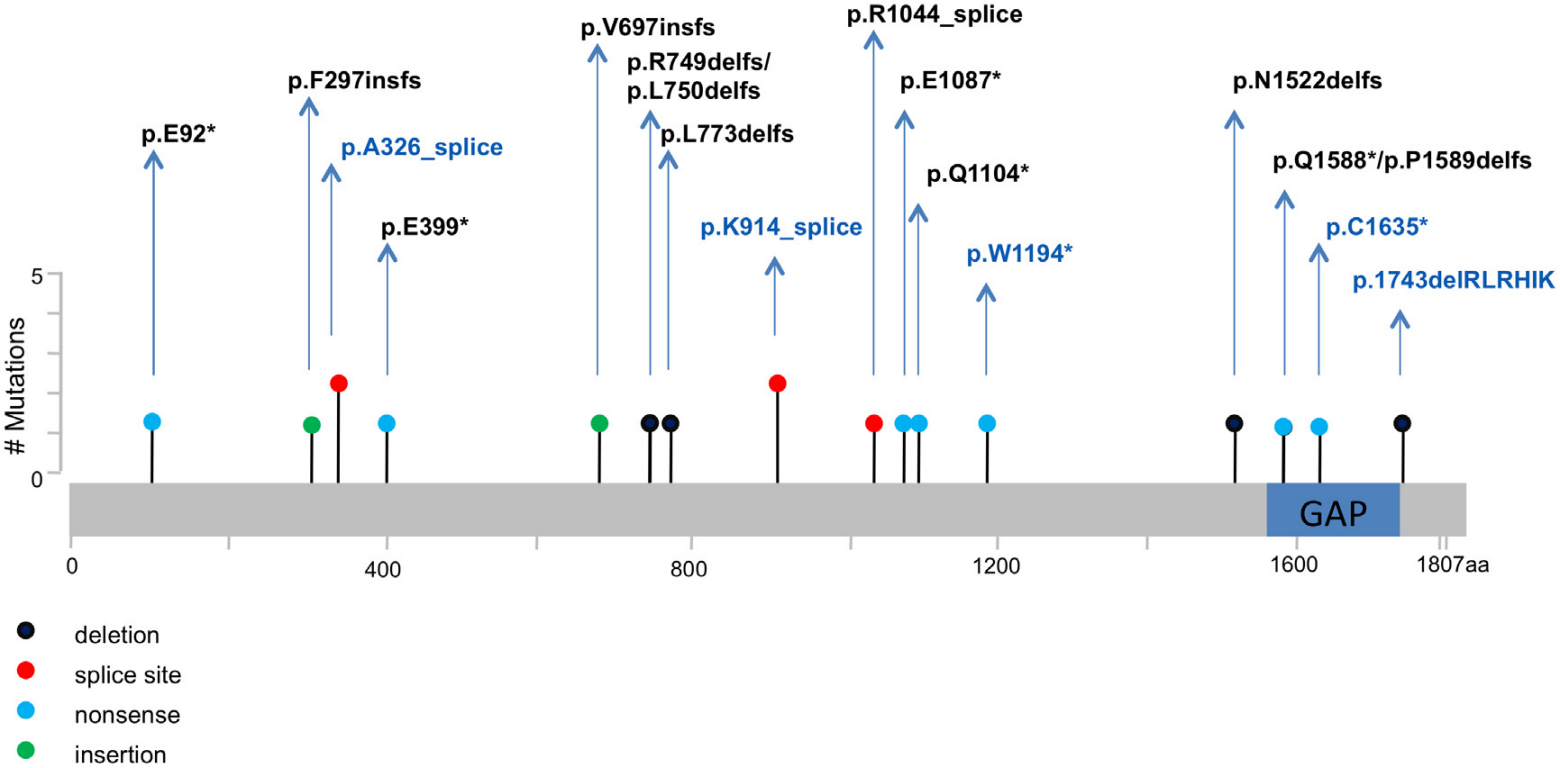
Map of 20 somatic *TSC2* mutations detected in angiomyolipoma and LAM specimens. The GAP domain of TSC2 is indicated. Novel variants (n = 13) are in black font; variants previously reported (n = 5) are in blue font. Circle colors reflect different types of mutation, as indicated. Note that in two instances, the mutations differ by a single amino acid position, and hence their circles overlap (p.R749delfs/p.L750delfs, p.Q1588*/p.P1589delfs). Two mutations were seen twice each in two samples.

Inactivating mutations in *TSC2* were found in 30 of 32 (94%) tumors from 14 of 15 patients (Table 1). The remaining 2 tumors (6%, S30 and S31) were from one patient (P14), who had a germ line *TSC1* nonsense mutation (c.2074C>T; p.R692*). Among the 22 somatic mutations in *TSC2*, there were 6 small deletions (4 single nucleotide, one 18 nucleotide), 5 splice site mutations, 7 nonsense mutations, 2 small insertions (1 nucleotide each), and 2 large-scale deletions that removed the entire gene in one sample (S7) (homozygous deletion region 50kb, single copy loss region 150kb) (Fig 2). Apart from the absence of missense mutations, this mutation distribution is similar to that for *TSC2* germ-line mutations [20]. See S1 Table for a glossary of genetic terms used in this publication.

Of the 11 angiomyolipoma samples from subjects without TSC, six (S6, S8, S9, S10, S11, S12) had two distinct *TSC2* somatic mutations, indicative of biallelic inactivation. One sample (S7) showed a homozygous somatic deletion (50kb) of *TSC2*, as noted above, confirmed by multiplex ligation-dependent probe amplification (MLPA) and CapSeg analysis (S2 Fig) [21]. The remaining 4 of 11 non-TSC angiomyolipoma samples (S3, S4, S13, S32) showed copy neutral LOH. The chylous fluid and abdominal LAM samples (S1, S2) from patient P1 who had sporadic LAM, had the same *TSC2* single nucleotide mutation (c.2319delA, p.L773delfs) and copy neutral LOH. Thus all 13 sporadic angiomyolipoma and LAM specimens showed evidence of biallelic TSC2 inactivation.

Analysis of the 16 renal angiomyolipomas from one patient (P13), who has TSC, revealed a non-mosaic germline *TSC2* deletion mutation (c.5135delC) that was also seen in normal tissue (Table 1). Two tumor samples (S26, S27) from this subject had *TSC2* somatic “second hit” mutations. Twelve of the remaining 14 angiomyolipomas showed *TSC2* copy neutral LOH, while neither second hit mutation nor LOH was detected in two of 16 samples (S16, S28) (Table 1).

**Fig 3:**
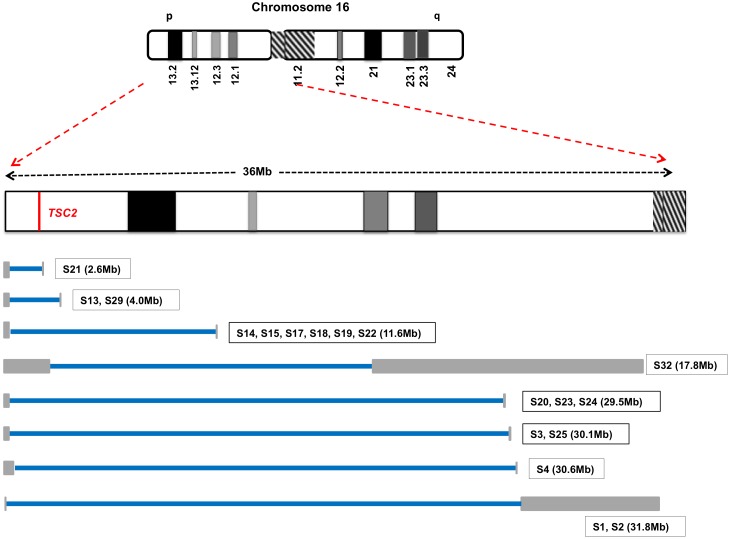
Copy neutral-LOH on chromosome 16p. At least 8 different regions of copy neutral LOH were seen in 18 tumor samples with 16p LOH. The blue bars reflect the region of copy neutral LOH for each sample extending from the first to the last SNP with skewed allele frequency (AF <0.4 or >0.6). The gray bars represent the interval between the last SNP with normal AF (0.4 < AF < 0.6) and the first SNP with skewed AF on each side of the region with LOH, and reflect regions with no informative SNP markers to assess LOH.

LOH mapping using informative SNPs for 18 different samples from 6 patients permitted the delineation of at least 8 different regions of chromosome 16p LOH (Fig 3; S3 Fig). In all of these tumors the LOH region extended from the first SNP identified near 16pter to a region more centromeric on chromosome 16p, and encompassed *TSC2* at g.16:2,095,990–2,140,713 (hg19). The smallest region of LOH was ~ 3Mb, and the largest was ~ 30Mb. The largest LOH region included the centromeric region of chromosome 16 but none extended to include all of chromosome 16, suggesting that mitotic recombination had occurred somewhere on the p arm or centromere in each case to replace the wild type *TSC2* locus with the pathogenic mutation. Note that this mechanism preserves diploid copy number for all other genes on 16p. Copy neutral LOH for all of 9q, including *TSC1* at g.9:135,764,735–135,822,020 (hg19) on 9q34, was seen in the two samples (S30, S31) from patient P14 with the germline *TSC1* p.R692* mutation. Copy number analysis using ABSOLUTE [22] also identified a subclonal, 2.4-fold cancer cell fraction copy number for chromosome 9q in sample S15, although the target of this event is unknown. Apart from these alterations centered on TSC1 and TSC2, no other chromosomal gains or losses were identified in our cohort.

### Whole exome sequencing findings

Whole exome sequence analysis of 23 tumor normal pairs uncovered a median of 4 coding mutations per tumor (not including those identified in *TSC1*/*TSC2*, range 0–12), with 3 of 78 (4%) frame-shift deletions, 3 of 78 (4%) in-frame deletions, 2 of 78 (3%) nonsense, 2 of 78 (3%) splice site, and 68 of 78 (87%) missense mutations (Table 2). In addition, among the 68 missense mutations, 10 (15%) were neutral, 21 (31%) were of low potential, 31 (46%) were of medium potential, and 6 (9%) were of high potential for having functional effects according to Mutation Assessor [23, 24]. This corresponds to a mutation rate of 0.12 (range 0–0.36) non-synonymous mutations/Mb (excluding *TSC1*/*TSC2*). Three angiomyolipomas from P13 (S20, S23, and S24) had identical mutations in *MAATS1* and *NCF1* (Table 2), suggesting that they were derived from a single clone, as discussed in more detail below. Two samples from P13 had different mutations in *TRIP12* (S24 c.5470-2A>G, S27 p.R1595Q) (Table 2), suggesting that alteration of this gene may contribute to development of these tumors. *TRIP12* is an ubiquitin ligase for ARF, which is reported to suppress cell growth by activating p53 [25], and frequently harbors inactivating somatic point mutations (100 of 380 (26%) somatic mutations are inactivating [26, 27]) in diverse cancer types. However, although the p.R1595Q somatic mutation has been seen in a bladder cancer and a stomach cancer, the functional significance of that missense mutation is unknown.

**Table 2:** Somatic genetic alterations other than *TSC1*/*TSC2* identified in 23 angiomyolipoma/LAM samples subject to tumor-normal paired exome sequencing.

Patient #	Sample #	Gene	Variant Type	cDNA Change	Protein Change	Mutant allele fraction	Mutant Read count	Ref read count	Validation/Confirmation	Mutationassessor
P1	S1	*TENM2*	Missense	c.6241G>A	p.D2081N	0.232	36	119	WGS	low
		*B3GALT5*	Missense	c.673G>A	p.V225M	0.146	19	111	WGS	medium
		*MAZ*	Missense	c.1148C>T	p.A383V	0.462	6	7	WGS	neutral
		*GZF1*	Missense	c.1021G>A	p.G341S	0.215	23	84	RNA-seq	low
P1	S2	TENM2	Missense	c.6241G>A	p.D2081N	0.341	15	29	seen in S1	low
		*B3GALT5*	Missense	c.673G>A	p.V225M	0.293	60	145	seen in S1	medium
		*MAZ*	Missense	c.1148C>T	p.A383V	not seen	no coverage			neutral
		*GZF1*	Missense	c.1021G>A	p.G341S	0.261	30	85	RNA-seq	low
P2	S3	no mutations								
P3	S4	*ATP7B*	Missense	c.2173A>T	p.R725W	0.404	40	59	Sanger	medium
		*GPT2*	Nonsense	c.274C>T	p.R92*	0.400	46	69	Sanger	NA
P4	S5	*DBC1*	Missense	c.446G>A	p.R149H	0.275	11	29	RNA-seq	neutral
		*LSS*	Missense	c.1010C>T	p.P337L	0.333	31	62	RNA-seq	high
		*DSTYK*	Missense	c.865A>G	p.I289V	0.353	12	22	RNA-seq	medium
		*MCLN2*	Missense	c.596C>G	p.S199C	0.120	3	22	RNA-seq	medium
		*DYNC1H1*	Missense	c.8635A>G	p.K2879E	0.053	4	71	RNA-seq	medium
P5	S6	*CNOT3*	Missense	c.38G>A	p.R13H	0.182	32	144	RNA-seq	medium
		*KIAA1370*	Missense	c.832G>A	p.E278K	0.276	16	42	RNA-seq	low
		*ABCB4*	Missense	c.2339G>A	p.G780D	0.278	40	104	RNA-seq	high
		*VILL*	Missense	c.1879G>A	p.A627T	0.288	15	37	RNA-seq	neutral
		*EFEMP1*	Missense	c.1279C>T	p.R427W	0.304	21	48	RNA-seq	medium
		*TNK2*	Missense	c.804C>G	p.I268M	0.056	4	68	RNA-seq	medium
P6	S7	*SPEN*	Missense	c.5437G>A	p.A1813T	0.138	12	75	Sanger	neutral
		*SNRPA*	Missense	c.718G>C	p.V240L	0.100	9	81	RNA-seq	medium
		*SGOL2*	Missense	c.1433G>C	p.G478A	0.162	30	155	RNA-seq	low
		*CNIH3*	Missense	c.400G>A	p.E134K	0.364	40	70	RNA-seq	medium
		*GSK3B*	Splice_Site	c.1195_splice	p.D399_splice	0.293	12	29	RNA-seq	NA
		*ATP1A1*	Missense	c.1696C>T	p.P566S	0.080	16	183	RNA-seq	low
		*THBS3*	Missense	c.1576G>C	p.D526H	0.087	9	94	RNA-seq	medium
		*ZNF304*	Nonsense	c.688A>T	p.K230*	0.075	6	74	RNA-seq	NA
		*SEL1L2*	Missense	c.511G>A	p.E171K	0.128	6	41	RNA-seq	medium
		*ZNF592*	Missense	c.3158C>G	p.P1053R	0.082	10	112	RNA-seq	neutral
		*THBS3*	Missense	c.1576G>C	p.D526H	0.087	9	94	RNA-seq	medium
		*TRPM6*	Missense	c.3965C>T	p.T1322I	0.164	31	158	RNA-seq	low
P13	S14	*GIGYF1*	Missense	c.953G>T	p.G318V	0.227	17	58	Sanger	neutral
		*NEUROD4*	Missense	c.704C>T	p.S235L	0.183	28	125	Sanger	low
		*POLR3F*	Missense	c.321A>G	p.I107M	0.149	7	40	Sanger	medium
P13	S15	*TMPPE*	Frame_Shift_Del	c.139_143delCAGCT	p.QL47fs	0.119	77	570	Sanger	NA
		*KIAA0232*	Missense	c.1672A>G	p.M558V	0.195	160	660	Sanger	low
		*ANK2*	Missense	c.7060C>T	p.R2354C	0.233	179	590	Sanger	neutral
		*PHF1*	Missense	c.160G>T	p.V54L	0.221	147	518	Sanger	low
		*UBE3D*	Missense	c.344G>T	p.G115V	0.171	118	571	Sanger	medium
		*OR8G5*	Missense	c.931G>T	p.V311L	0.155	162	886	Sanger	low
		*ANKRD35*	Missense	c.200C>A	p.T67K	0.088	58	603	SNaPshot	neutral
		*ZNF804B*	Missense	c.643C>T	p.H215Y	0.057	48	799	SNaPshot	medium
P13	S16	no mutations								
P13	S17	*WWTR1*	Missense	c.317C>T	p.P106L	0.841	37	7	Sanger	medium
		*NDUFA8*	Missense	c.284A>G	p.Q95R	0.847	294	53	Sanger	low
		*GCAT*	Missense	c.1201G>T	p.G401W	0.885	69	9	Sanger	high
		*ARHGAP35*	Missense	c.3818A>C	p.E1273A	0.782	68	19	Sanger	high
		*CCDC132*	Missense	c.2460A>C	p.L820F	0.882	45	6	Sanger	medium
P13	S18	*MLKL*	Missense	c.553A>G	p.M185V	0.208	10	38	Sanger	low
		*USH2A*	Missense	c.9638C>A	p.P3213Q	0.221	73	258	Sanger	medium
		*CCDC39*	Missense	c.892C>T	p.R298C	0.326	29	60	Sanger	NA
		*LIPA*	Missense	c.739G>A	p.V247I	0.133	33	216	Sanger	neutral
		*ZNF821*	Missense	c.518C>G	p.S173W	0.258	54	155	Sanger	low
		*MIER2*	Missense	c.1514C>T	p.S505L	0.187	46	200	Sanger	low
		*GPR50*	Missense	c.1214A>G	p.K405R	0.092	22	218	SNaPshot	low
		*TUBB2A*	In_Frame_Del	c.89_103delTCGACCCCACAGGCA	p.IDPTG30del	0.090	7	71	Sanger	NA
P13	S19	no mutations								
P13	S20	*ADAMTSL1*	In_Frame_Del	c.1387_1398delTGCATCGACCAT	p.CIDH463del	0.099	19	172	Sanger	NA
		*ZSCAN21*	In_Frame_Del	c.1225_1239delCTCCACACCGGAGAG	p.LHTGE409del	0.097	10	93	Sanger	NA
		*MAATS1*	Missense	c.743G>A	p.R248H	0.295	33	79	Sanger	medium
		*NCF1*	Missense	c.568G>A	p.E190K	0.274	51	135	Sanger	medium
P13	S21	*UBE3D*	Frame_Shift_Del	c.1130_1131delGC	p.R378fs	0.087	12	126	SNaPshot	NA
P13	S22	no mutations								
P13	S23	*KIAA1107*	Missense	c.334G>A	p.V112I	0.222	8	28	Sanger	neutral
		*HOXD12*	Missense	c.491A>G	p.D164G	0.125	25	175	Sanger	medium
		*BAP1*	Missense	c.1550C>T	p.T517M	0.126	23	160	Sanger	low
		*TLL1*	Missense	c.1120C>T	p.H374Y	0.169	44	216	Sanger	low
		*MAATS1*	Missense	c.743G>A	p.R248H	0.247	20	61	Sanger	medium
		*NCF1*	Missense	c.568G>A	p.E190K	0.236	60	194	Sanger	medium
		*ATP9A*	*Missense*	c.2813T>G	p.I938S	0.222	8	28	SNaPshot	medium
P13	S24	*ASPM*	Missense	c.2296C>T	p.R766C	0.271	97	261	SNaPshot	medium
		*TRIP12*	Splice_Site	c.5470-2A>G	p.L1824_splice	0.254	36	106	Sanger	NA
		*MIS18BP1*	Missense	c.816C>A	p.S272R	0.333	54	108	Sanger	low
		*ZNF578*	Frame_Shift_Del	c.1414_1417delAGAC	p.RH472fs	0.241	27	85	Sanger	NA
		*MAATS1*	Missense	c.743G>A	p.R248H	0.380	30	49	Sanger	medium
		*NCF1*	Missense	c.568G>A	p.E190K	0.267	60	165	Sanger	medium
		*ASPM*	Missense	c.247C>G	p.P83A	0.081	33	374	Sanger	medium
P13	S25	no mutations								
P13	S26	*WWC1*	Missense	c.2896C>T	p.R966C	0.352	51	94	Sanger	medium
P13	S27	*TRIP12*	Missense	c.4784G>A	p.R1595Q	0.216	19	69	Sanger	high
		*WDR55*	Missense	c.790G>A	p.E264K	0.466	54	62	Sanger	medium
		*LILRB5*	Missense	c.1291G>A	p.D431N	0.106	7	59	Sanger	low
		*EXOC3*	Missense	c.1975A>G	p.I659V	0.074	12	150	SNaPshot	low
P13	S28	no mutations								
P13	S29	*PTPRF*	Missense	c.2269C>G	p.R757G	0.030	6	193	Sanger	medium
		*PDE2A*	Missense	c.930C>A	p.D310E	0.122	22	159	SNaPshot	medium
		*RNF24*	Missense	c.266A>G	p.D89G	0.329	69	141	Sanger	low
		*GRM3*	Missense	c.2498A>G	p.N833S	0.079	15	174	SNaPshot	medium
		*POLR2A*	Missense	c.2423C>G	*p*.*P808R*	0.070	15	198	SNaPshot	high

WGS, whole genome sequencing.

Apart from *MAATS1*, *NCF1*, and *TRIP12*, no other genes were mutated in more than one tumor. Gene set enrichment analysis (GSEA) showed no enrichment of genes with mutations within hallmark gene sets, computational gene sets (including cancer gene neighborhoods, and cancer modules), GO gene sets (including GO cellular component, molecular function and GO molecular function gene dataset), or oncogenic signatures genesets [28] [29]. Furthermore, KEGG and Protein Interaction Network Module analyses found no enrichment of genes with mutations within any biological pathway or functional gene network [30] [31, 32]. Next, we examined each mutated gene for its potential genetic or physical interaction with *TSC2* or *TSC1*, using BioGRID, Coremine, and Esyn databases. Only *GSK3B* was identified as an interactor with *TSC2*, based on their common involvement in PI3K-AKT-mTOR signaling [33] [34].

Singleton mutations were found in 3 of the 263 genes recurrently mutated across cancer as annotated by the ‘PanCan’ dataset [35, 36]: *BAP1* (p.T517M in S23 from P13), *ARHGAP35* (p.E1273A in S17 from P13), and *SPEN* (p.A1813T in S7 from P6) (Table 1). The *BAP1* mutation has been previously seen in two cancer cell lines, derived from a Ewing sarcoma, and a lung adenocarcinoma [26, 27]. The other two mutations have not been reported previously [26, 27]. All are missense changes of uncertain functional effect.

In a search for potential structural rearrangements affecting these tumors, we performed whole genome sequencing of two tumor-normal pairs (S1 and S3). However, there was no evidence for genomic rearrangement in these samples using dRanger [37], and no new exonic mutations were identified by this analysis beyond the previous whole exome analysis.

### Phylogenetic analysis of the 16 angiomyolipomas from TSC patient P13

**Fig 4:**
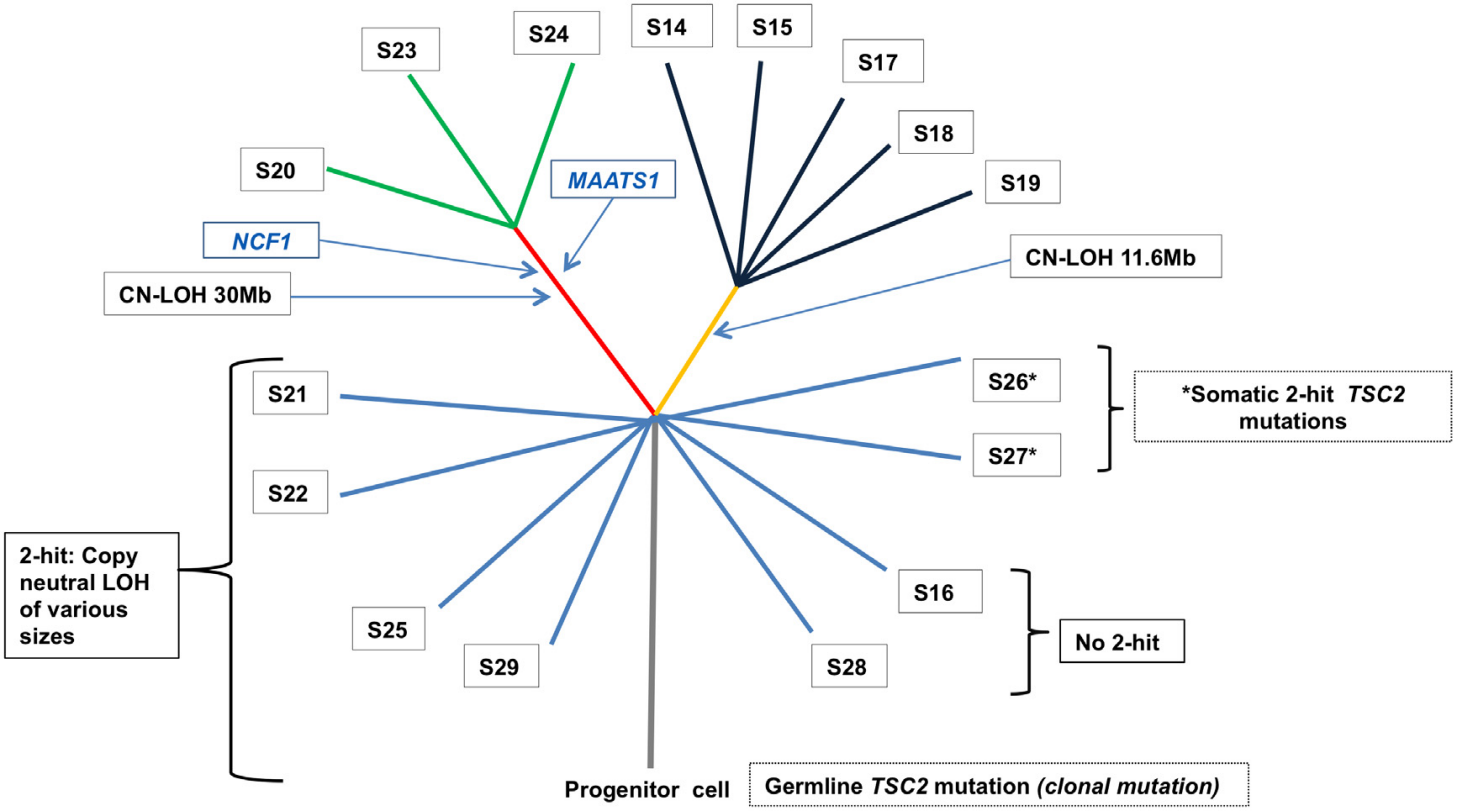
Phylogenetic tree of 16 angiomyolipomas from one TSC patient. A model of angiomyolipoma development from a progenitor cell in the kidney is shown, including 3 tumors (S20, S23, S24) that had a definite common truncal precursor, and 5 tumors (S14, S15, S17, S18, S19) that may have had a common truncal precursor. Note that the extent of copy neutral LOH in S22 is uncertain due to low tumor purity. CN-LOH denotes copy neutral LOH.

LOH mapping and somatic mutations were used to develop a phylogenetic tree for the 16 renal angiomyolipomas from P13 (Fig 4). There were 5 distinct regions of LOH on 16p in these tumors (Fig 3, S3 Fig). Samples S20, S23, and S24 all had the same region of chromosome 16 LOH, and identical mutations in *NCF1* and *MAATS1*, but also had unique mutations not seen the other two samples (Table 2). These observations suggest that these three tumors arose from a common precursor cell, sustaining these two early mutations prior to clonal expansion, followed by acquisition of additional mutations after dispersion (Fig 4). Five other tumors, S14, S15, S17, S18, S19, also had identical regions of LOH to the resolution limits of exome sequencing. However, these tumors did not share any sequence mutations, suggesting that mutations were acquired later in tumor development, or that they are not of common clonal ancestry. Five tumors, S21, S25, S26, S27, S29, had either unique regions of LOH (n = 3) or unique second hit *TSC2* point mutations (n = 2), indicating that each was clonally distinct. Two angiomyolipomas (S16 and S28) from this patient had no evidence of *TSC2* LOH, nor any confirmed somatic mutations, suggesting alternate mechanisms of *TSC2* loss not detected by our analysis, or potential low tumor purity. Sample S22 also had low tumor purity (~10%) but showed evidence of *TSC2* LOH without somatic mutations.

## Discussion

Consistent with extensive previous data and Knudson’s two-hit hypothesis [38], we identified biallelic inactivation of *TSC2* or *TSC1* in 30 of 32 angiomyolipomas and LAM tumors including point mutations, small indels, large genomic deletions, and copy neutral LOH. Interestingly, biallelic small mutations were more common in subjects without TSC (7 of 13, 54%) than in subjects with TSC (3 of 19, 12%; p = 0.05 Fisher exact test). Copy neutral LOH events were much more common in those with TSC. Using SNP allele frequency from our sequencing data, we were able to localize the site of aberrant cross-over events on 16p, and determine that at least 8 different regions were sites of mitotic recombination that led to copy number neutral LOH of *TSC2*.

We found that angiomyolipomas have very few non-synonymous exonic mutations (4 on average), and almost no recurrently mutated genes beyond *TSC1* and *TSC2*. Although a number of genes with singleton mutations in this data set are mutated in other cancers, we suspect that most are background non-functional mutations that happened to be present in the initiating cells of these tumors, and are not tumor driver events. In support of this notion, 31 of 82 (38%) of the mutations appeared to be subclonal with variant AF < 0.15, and 20 of 82 (24%) had variant AF < 0.1 (Table 2). In addition, 87% were missense mutations, and of those 46% were neutral or had low potential for functional effects. Furthermore, we found no aggregation of the genes with mutations in any particular pathway using multiple statistical approaches and candidate gene sets. While it is possible that the mutations in *ARHGAP35*, *BAP1*, and *SPEN* are significant, all were missense mutations and the functional effects are uncertain. Two mutations in *TRIP12* were also seen in different tumors, and while one is located in a splice site, the second (p.R1595Q) is of unknown functional effect.

Given its role in the mTOR signaling pathway [39], the singleton loss-of-function mutation in *GSK3B* (c.1195+1G>A, p.D399fs seen at clonal AF, 0.293, in S7) is arguably the strongest candidate to be a secondary driver mutation in this cohort. However, further study is required to examine the functional effect of this mutation in angiomyolipoma development.

Our data provide important information on clonality and tumor development for this class of tumors. Comparison of an abdominal LAM mass and a LAM cell cluster isolated from chylous fluid of a single patient (P1) revealed identical mutations in *TSC2* and somatic alterations in other genes indicating a common clonal origin, and no candidate genetic event to account for metastasis and survival of the LAM cell cluster. Analysis of 16 angiomyolipomas (S14 –S29) derived from one patient (P13) identified at least 7 tumors with independent clonal origin with unique regions of 16p copy neutral LOH or unique *TSC2* somatic mutations. These data strongly support the concept that the multiple angiomyolipomas that develop in most TSC adults are due to independent second hit mutations affecting *TSC2* or *TSC1* occurring in distinct progenitor cells, similar to what we have previously reported in TSC-associated RCC [40].

In P13, we also found evidence that single clones can seed multiple tumour masses, as 3 angiomyolipomas shared a common region of 16p LOH as well as two identical somatic mutations. These tumours each contained a small set of unique somatic mutations, indicative of further, independent clonal evolution. In addition, five tumor samples had the same region of 16p copy neutral LOH as well as other unique somatic mutations, and may or may not have been derived from a common precursor cell that became dispersed during tumor development. We also note that 3 of 16 angiomyolipomas from P13 had no somatic mutation findings, including two without second hit mutations in *TSC2*. Although these angiomyolipomas may be completely silent at the genomic level, it is also possible that the samples analyzed had a low tumor representation masking identification of both LOH and somatic mutations, or that epigenetic or other types of cancer genome variation underlie these tumors.

Our findings of the low somatic mutation rate of angiomyolipoma contrast sharply with cumulative cancer genome sequencing studies that have shown that adult malignancies have a wide range of non-synonymous mutations per cancer, ranging from 8 (chronic lymphocytic leukemia) to over 1,000 (colorectal cancer with microsatellite instability; other cancers with *POLE* mutations) [35, 41]. Angiomyolipoma, with a median of 4 non-synonymous mutations per tumor (range of 0 to 12) is well below the range of these common cancer types. However, infant and pediatric malignancies have a much lower mutation rate in general with a median of 4 to 13 among 5 pediatric cancers (acute lymphoblastic leukemia (ALL), medulloblastoma, neuroblastoma, glioblastoma, and rhabdoid cancers) [41]. In addition, several infant or pediatric malignancies appear to be due to a single genetic event: *MLL-AF4* fusion in infant acute lymphoblastic leukemia [42], homozygous inactivation of *SMARCB1* in pediatric rhabdoid tumors [43], and *C11orf95-RELA* fusion in brain ependymoma [44]. As noted by others [45], each of these three mutations affect chromatin remodeling genes that may be predicted to have wide-ranging effects on gene transcription, driving tumor development and growth in a pleiotypic manner. In addition, these singleton genetic events are seen in unique and rare malignancies likely derived from specific cell types at specific developmental stages of early childhood, suggesting that each (different) cell of origin is sensitive to the global chromatin and transcriptional regulatory derangements induced by the singleton genetic event.

Our findings suggest that angiomyolipomas fit this general model of pediatric tumor development, with few somatic mutations and a single critical target of inactivating mutation (in the case of angiomyolipoma, this is usually *TSC2* and less commonly *TSC1*) that initiates and drives tumor development. Consistent with this model is the observation that small angiomyolipomas can be seen in the kidneys of young children with TSC and are present in up to 80% of TSC children by the age of 10 [46]. In addition, these tumors are typically indolent, growing slowly over a number of years, and usually do not require surgical intervention or other treatment, with a slowing in tumor growth in older adults. Furthermore these observations suggest that there is likely a specific cell type (currently unknown) that is resident in the early kidney that is sensitive to the tumor and growth promoting effects of *TSC2* or *TSC1* loss. Loss of *TSC2* or *TSC1* results in unregulated activation of mTORC1 with wide-ranging signaling, metabolomic, and transcriptional effects [39, 47, 48], which likely enable this singleton transforming event. In addition, there may be other clinically important consequences of TSC protein complex loss beyond mTORC1 activation that are not well understood.

Very limited similar studies have been performed for tumors occurring in other tumor suppressor gene syndromes with low growth potential. Whole exome sequencing of 7 neurofibromas from a patient with Neurofibromatosis type I led to identification of second hit mutations in NF1 in 5 of 7 tumors, and median of 0 (maximum 1) non-synonymous mutations in the rest of the exome [49]. Whole exome sequencing of 4 renal cell carcinomas (RCC) occurring in a 32 yo man with Von Hippel Lindau syndrome led to identification of unique second hit mutations in *VHL* in each RCC, as well as 13–23 other somatic non-synonymous mutations throughout the genome [50]. This increased level of somatic mutation in VHL RCCs is consistent with the more malignant behavior of these tumors compared to angiomyolipoma, but is less than that seen in RCC in those without VHL syndrome [50].

The remarkably low mutation rate and genomic stability observed in angiomyolipoma is consistent with continuing long-term responses (> 5 years in some cases) of these tumors to treatment with rapamycin and related drugs that inhibit mTORC1[51]. However, it is notable that that complete responses are virtually unknown and even partial responses, according to RECIST criteria, are rare.

## Materials and Methods

### Ethics statement

Informed written consent was obtained from all patients whose samples were studied. The study was approved by the Partners Human Research Committee of Brigham and Women’s Hospital (2007P000699, 2010P001818).

### Patient samples

Thirty-two samples, comprising 30 fresh resected tumor tissues, one chylous pleural fluid sample, and one short-term culture from a resected angiomyolipoma, were collected from 15 patients with angiomyolipoma (n = 11) or concurrent angiomyolipoma and LAM (n = 4) (Table 1). All samples were collected from patients undergoing surgery as part of routine clinical care, and had been seen either at one of our hospitals (Brigham and Women’s Hospital or Massachusetts General Hospital), or in one case was referred from another institution. Normal adjacent tissue, saliva, and/or blood were also collected as control samples for 24 of the samples.

### Sample processing

Genomic DNA was extracted from fresh frozen tumor specimens using Puregene DNA Purification Kit (QIAGEN, formerly Gentra/Puregene) according to the manufacturer’s protocol. The chylous fluid sample was subjected to filtering on a 40 μm nylon mesh (Cell Strainer from BD Biosciences). LAM cell clusters were collected and cultured in Lonza Clonetics SmGM-2 Smooth Muscle Growth Medium (containing 5% fetal bovine serum) in a humidified incubator at 5% CO_2_ [52]. Clusters of LAM cells were visually identified and a single large cluster was aspirated, and subjected to DNA extraction as described [52], and Phi29-based whole genome amplification using the GE Healthcare illustra GenomiPhi V2 DNA Amplification Kit.

### Deep coverage sequence analysis of *TSC1* and *TSC2*

To identify mutations in *TSC1* and *TSC2* with high sensitivity, we performed targeted sequence analysis of long-range PCR products at high read depth (>500x) as described previously [53]. This assay was used on the first six samples (subject P1-P6). Sequencing data output was analyzed using the Genome Analysis Toolkit [54], as well as custom software as described [53] to enable the detection of all sequence variants at allele frequency ≥1%.

### Whole exome and genome sequencing

Exome sequencing was performed by the Broad Institute Genomics Platform and analyzed using a standard analytic pipeline deployed in the Firehose environment. Exome capture targeted 33 Mb in 193,094 exons in 18,863 genes [55]. Briefly, reads were aligned using bwa, followed by indel realignment and quality score recalibration using the Genome Analysis Toolkit [54]. Somatic mutations were identified from tumor-normal pairs using MuTect [56] and Indelocator [57]. This sequence data was also examined for mutations in *TSC1* and *TSC2* using custom software as described [53] to enhance detection of sequence variants. For the first 13 samples (Table 1), the median read depth was 86x for the targeted region, with median of 89% of target bases having a read depth of ≥ 20x, and median of 70% of target nt having a read depth of ≥ 50x. For the latter 19 angiomyolipoma samples (Table 1), median read depth was 191x for the targeted region, with median of 95.5% of target nt having a read depth of ≥ 20x, and median of 90% of target nt having a read depth of ≥ 50x.

Whole genome sequencing (WGS) was also performed at the Broad Institute following similar methods, with median read depth of 33x. Sequence variants were detected using the tools noted above and structural rearrangements were detected using dRanger [37].

### Copy number changes and LOH mapping

Copy number profiles were derived from fractional coverage values of each exon compared with a panel of normals using Cap-Seg [58] and Allelic-CapSeg in the Clonal Evolution Exome Suite in the Firehose environment. Somatic LOH was quantified using ABSOLUTE [22] applied to variant and copy number calls derived from exome sequence data.

Detailed LOH mapping was performed for the paired tumor samples by: 1) identification of heterozygous SNPs in the normal sample using the following filters: read depth > 19, and variant AF between 0.40 and 0.60; and 2) determination of the variant AF for these SNPs in the tumor sample. In unpaired tumor samples, likely SNPs were identified by filtering for variants with variant AF between 0.05 and 0.95. Allele frequencies for these SNPs for each sample were graphed according to nucleotide position using Excel.

### Review of variant calls

All sequence variants identified by Mutect and Indelocator were reviewed. Those identified in intronic and intergenic regions, and in ncRNA; or with a total number of variant reads < 3 or reads present only in one direction; or those seen at an allele frequency of < 5% were not considered further. Remaining variants were reviewed manually using Integrative Genomics Viewer (IGV) [59, 60], and were examined in multiple samples including the tumor and normal control. Artifacts, misaligned reads, and synonymous variants were discarded. The remaining variants were validated by one of three methods. First, both whole exome and whole genome sequencing was performed on two samples (S1 and S3, Table 1), and variants seen concordantly by the two analyses were considered confirmed. Second, RNA-Seq was performed on 5 samples (Table 1), and variants seen in both whole exome sequencing and RNA-Seq analyses were also considered confirmed (full details on RNA-Seq will be reported elsewhere). Third, variants were confirmed by Sanger sequencing for those seen at > 10% allele frequency, and by SNaPshot single nucleotide sequencing for those seen at allele frequency > 5% and < 10%. Sanger and SNaPshot extension products were analyzed on an ABI 3100 sequencer (Applied Biosystems, Carlsbad, CA, USA). SNaPshot experiments were run in duplicate.

### Pathway analysis

Integrated pathway analysis was performed for genes in which somatic mutations were detected in two ways: one analysis for mutations found in the 16 distinct tumors from P13, and another for all somatic mutations found in 15 subjects. We used Gene Set Enrichment analysis (GSEA) [28, 29] and WebGestalt [31, 32] tools to search for pathway enrichment among the genes with somatic mutations in these tumors. We also explored each mutant gene using BioGRID, Coremine, and Esyn, to examine potential interactions between each gene and TSC2/TSC1 [33, 34]. We also searched COSMIC [61, 62], cBioPortal for Cancer Genomics [26, 27], that currently contains data from 105 cancer genomics studies and TumorPortal [36] to assess whether each of the identified genes and confirmed mutant variants had been seen previously in any type of cancer.

## Supporting Information

S1 Fig Kidneys resected from TSC patient P13 due to massive angiomyolipoma involvement. Left kidney had weight 1.465 kg and size 28.4 x 11.9 x 11.5 cm, and right kidney had weight 0.515 kg and size 15.6 x 10.8 x 5.4 cm.
(DOCX)

S2 Fig Homozygous genomic loss of *TSC2* in sample S7. A. MLPA graph. Top, analysis of S7 with a probe set covering *TSC2*. Control probes from other genomic sites have values from 0.81–1.25, while probes from *TSC2* have values from 0.29 to 0.45, indicative of homozygous loss with some normal cell contamination. A control sample is shown at bottom. Note that 05ex refers to exon 5 of *TSC2*, etc. Probes are sorted by size. **B. Capseg analysis visualized using IGV.** A 199 kb region surrounding *TSC2* is shown in IGV. Note that samples S4, S5, S6 show no copy number loss, while sample S7 shows homozygous copy number loss—dark blue, 0.42 copies—in a 50kb region; and single copy loss—light blue, 1.47 copies—in flanking regions. Gray regions are those with indeterminate copy number due to low coverage; white regions have copy number of 2.
(DOCX)

S3 Fig LOH Mapping in renal angiomyolipoma and LAM tumor samples. Graphs of allele fractions are shown for SNPs on chromosome 16 (Samples S1 –S29, S32) and chromosome 9 (Samples S30, S31).
(PPTX)

S1 Table Glossary of genetic terms used in this publication. (DOCX)
