# Supplementary material for: Whole Exome Sequencing Identifies TSC1/TSC2 Biallelic Loss as the Primary and Sufficient Driver Event for Renal Angiomyolipoma Development
Source: PLoS Genet. 2016 Aug 5;12(8):e1006242. doi: 10.1371/journal.pgen.1006242 (PMC4975391; doi:10.1371/journal.pgen.1006242)
Supplement: S1 Fig — Left kidney had weight 1.465 kg and size 28.4 x 11.9 x 11.5 cm, and right kidney had weight 0.515 kg and size 15.6 x 10.8 x 5.4 cm. (DOCX) [file pgen.1006242.s001.docx]

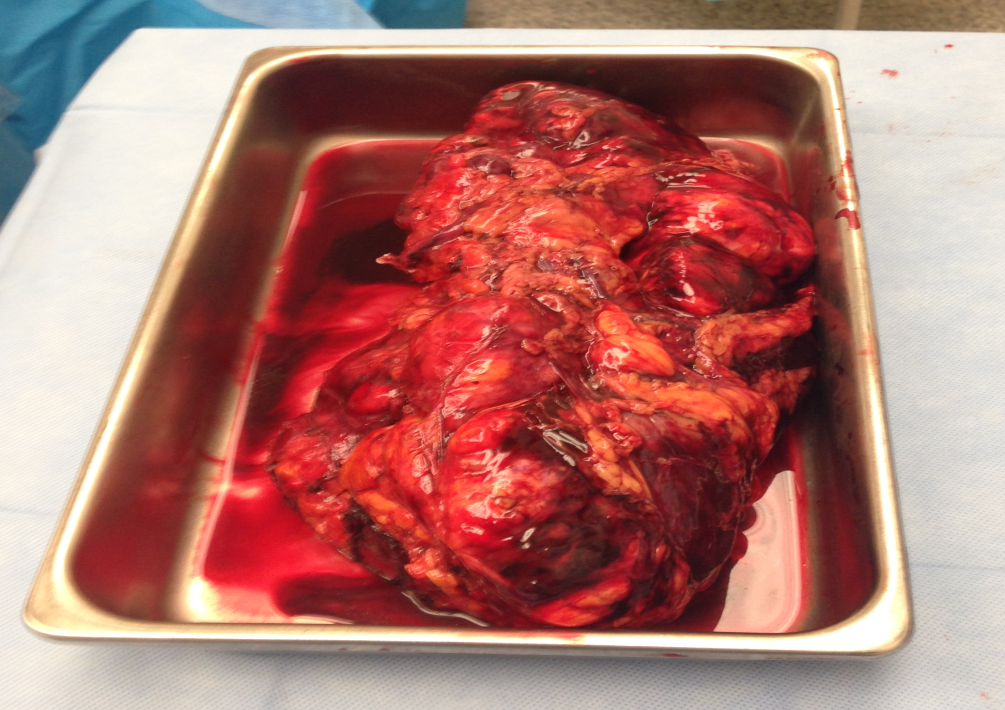

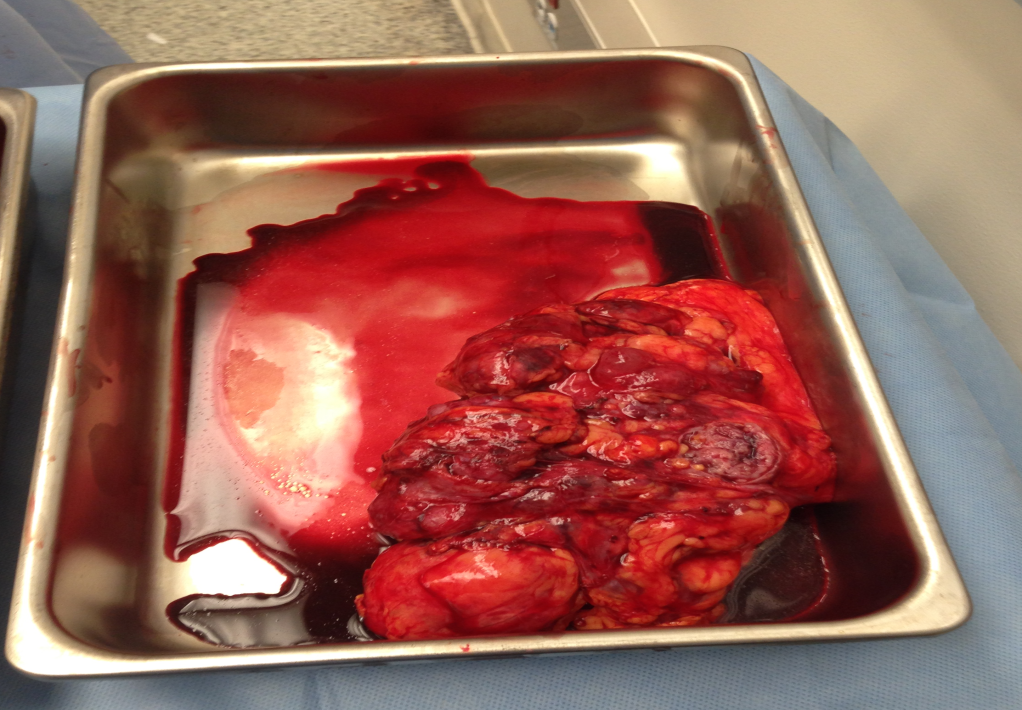


**S1 Figure. Kidneys resected from TSC patient P13 due to massive angiomyolipoma involvement.** Left kidney had weight 1.465 kg and size 28.4 x 11.9 x 11.5 cm, and right kidney had weight 0.515 kg and size 15.6 x 10.8 x 5.4 cm.
