# Supplementary material for: Whole Exome Sequencing Identifies TSC1/TSC2 Biallelic Loss as the Primary and Sufficient Driver Event for Renal Angiomyolipoma Development
Source: PLoS Genet. 2016 Aug 5;12(8):e1006242. doi: 10.1371/journal.pgen.1006242 (PMC4975391; doi:10.1371/journal.pgen.1006242)
Supplement: S2 Fig — Top, analysis of S7 with a probe set covering TSC2. Control probes from other genomic sites have values from 0.81–1.25, while probes from TSC2 have values from 0.29 to 0.45, indicative of homozygous loss with some normal cell contamination. A control sample is shown at bottom. Note that 05ex refers to exon 5 of TSC2, etc. Probes are sorted by size. B. Capseg analysis visualized using IGV. A 199 kb region surrounding TSC2 is shown in IGV. Note that samples S4, S5, S6 show no copy number loss, while sample S7 shows homozygous copy number loss—dark blue, 0.42 copies—in a 50kb region; and single copy loss—light blue, 1.47 copies—in flanking regions. Gray regions are those with indeterminate copy number due to low coverage; white regions have copy number of 2. (DOCX) [file pgen.1006242.s002.docx]

**A**

**
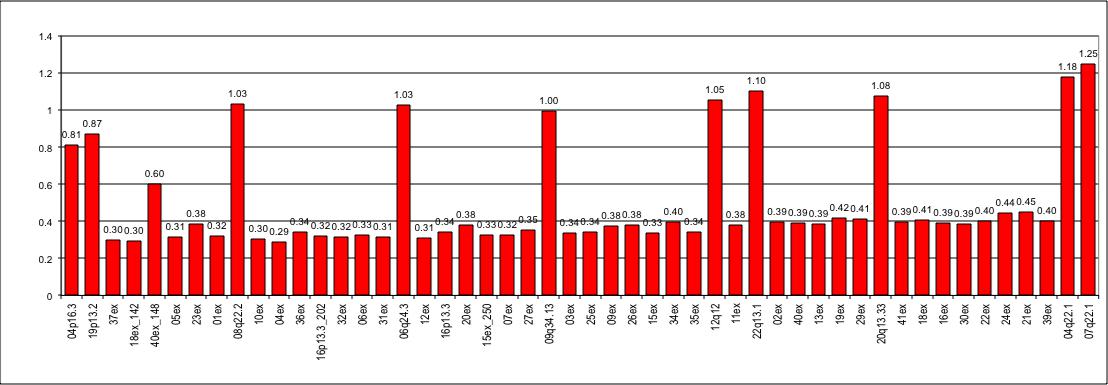
S7**

**Normal**


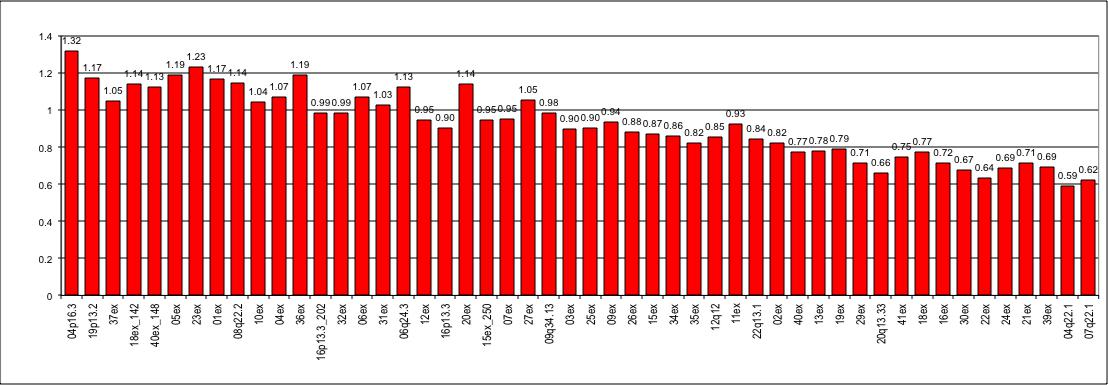


**B**

**
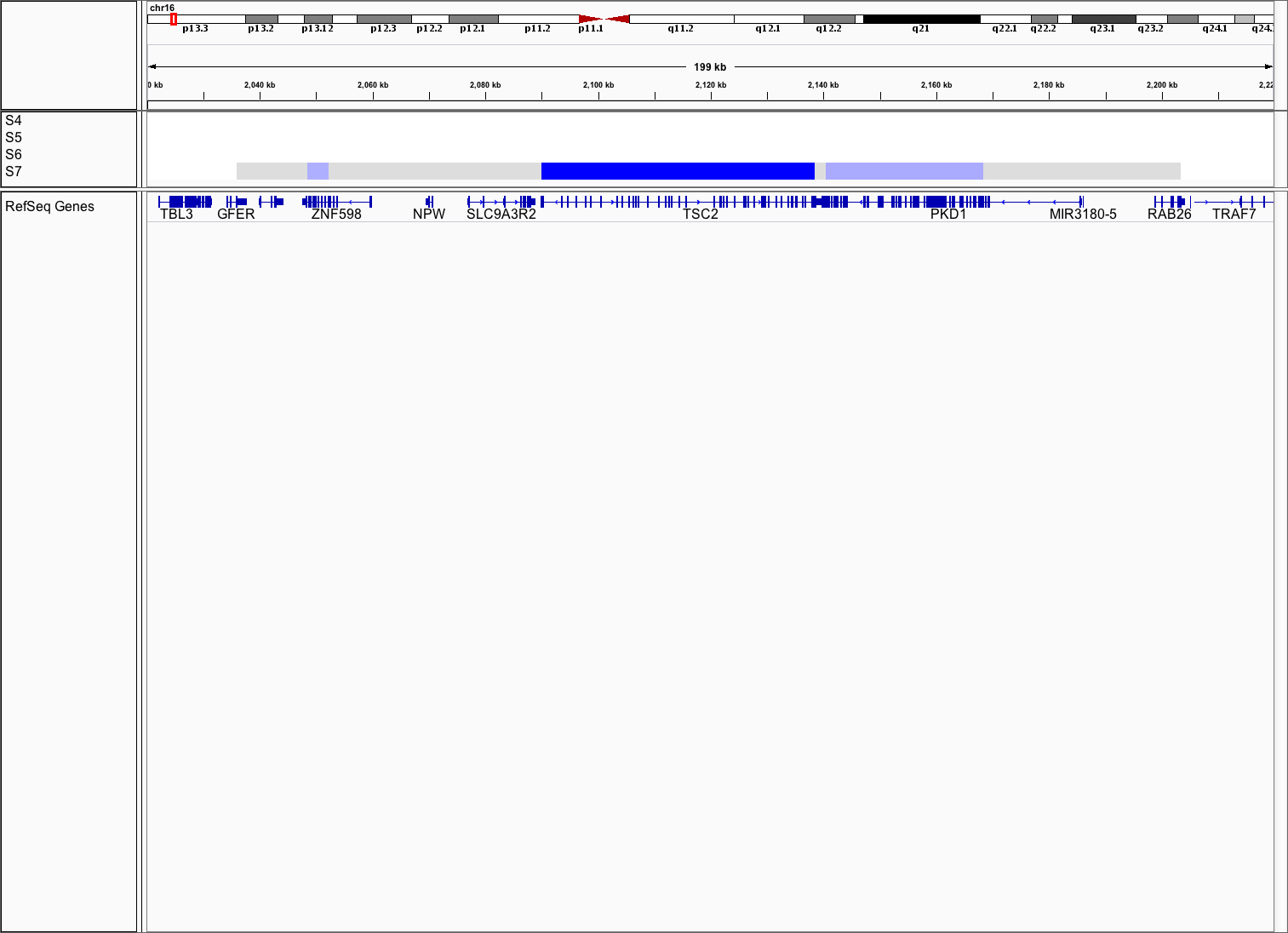
**

**S2 Figure. Homozygous genomic loss of *TSC2* in sample S7.**

**A. MLPA graph.** Top, analysis of S7 with a probe set covering *TSC2*. Control probes from other genomic sites have values from 0.81 – 1.25, while probes from *TSC2* have values from 0.29 to 0.45, indicative of homozygous loss with some normal cell contamination. A control sample is shown at bottom. Note that 05ex refers to exon 5 of *TSC2*, etc. Probes are sorted by size.

**B. Capseg analysis visualized using IGV.** A 199 kb region surrounding *TSC2* is shown in IGV. Note that samples S4, S5, S6 show no copy number loss, while sample S7 shows homozygous copy number loss – dark blue, 0.42 copies – in a 50kb region; and single copy loss – light blue, 1.47 copies – in flanking regions. Gray regions are those with indeterminate copy number due to low coverage; white regions have copy number of 2.
