# Supplementary material for: Whole Exome Sequencing Identifies TSC1/TSC2 Biallelic Loss as the Primary and Sufficient Driver Event for Renal Angiomyolipoma Development
Source: PLoS Genet. 2016 Aug 5;12(8):e1006242. doi: 10.1371/journal.pgen.1006242 (PMC4975391; doi:10.1371/journal.pgen.1006242)
Supplement: S3 Fig — Graphs of allele fractions are shown for SNPs on chromosome 16 (Samples S1 –S29, S32) and chromosome 9 (Samples S30, S31). (PPTX) [file pgen.1006242.s003.pptx]

## Slide 1
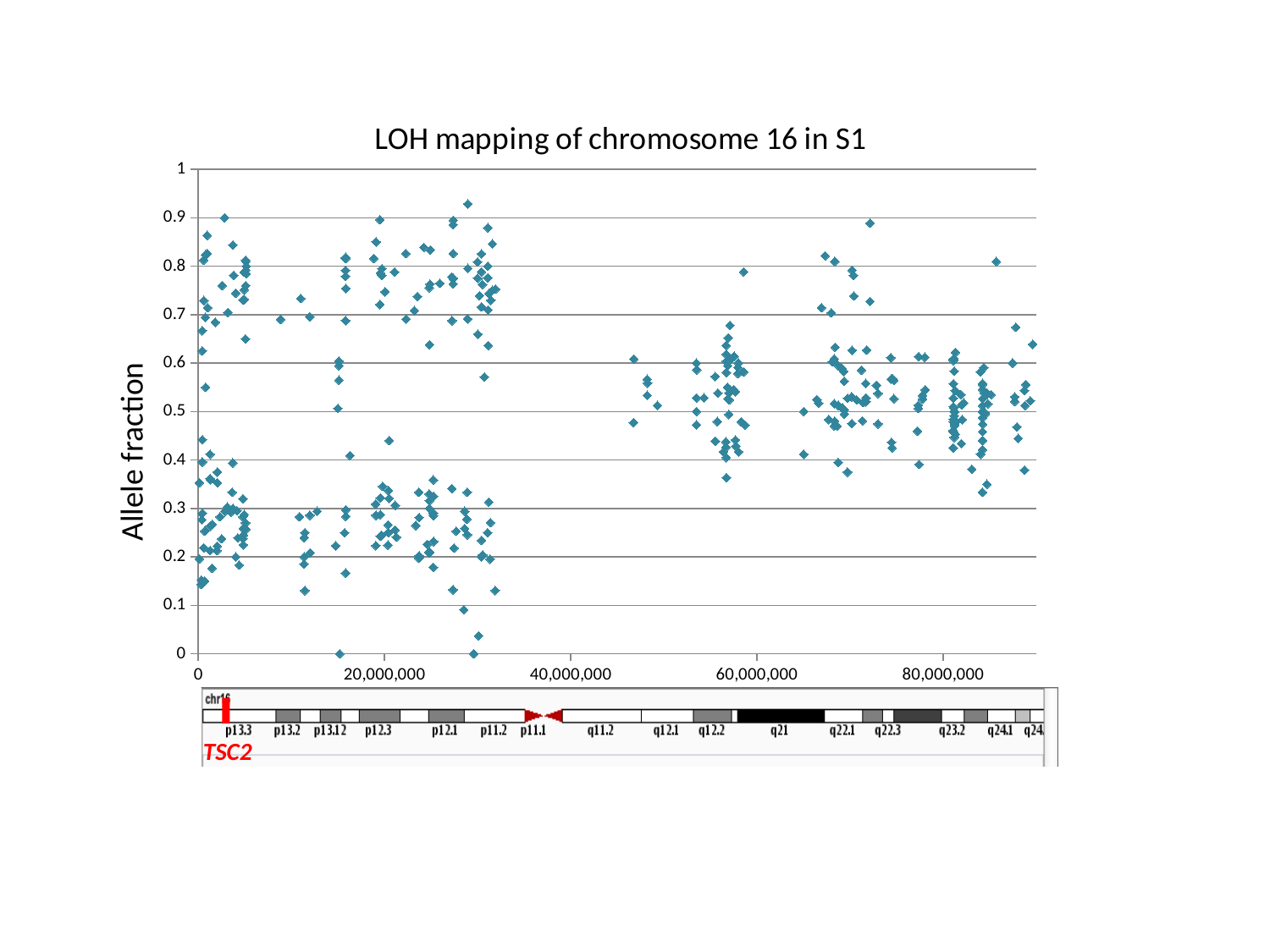

### Chart: LOH mapping of chromosome 16 in S1
| Category | t ratio |
|---|---|Allele fraction
TSC2

## Slide 2
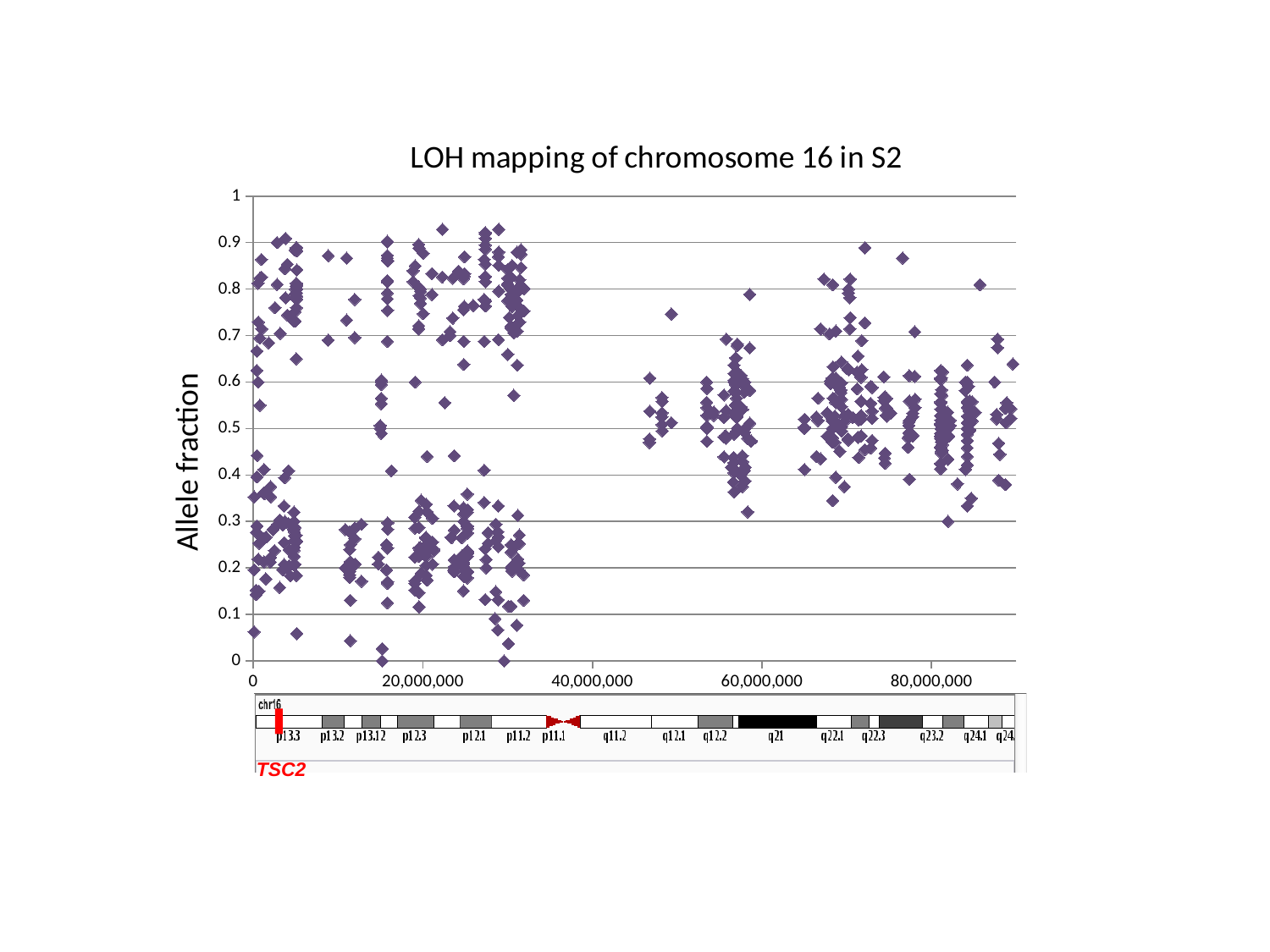

### Chart: LOH mapping of chromosome 16 in S2
| Category | t ratio |
|---|---|Allele fraction
TSC2

## Slide 3
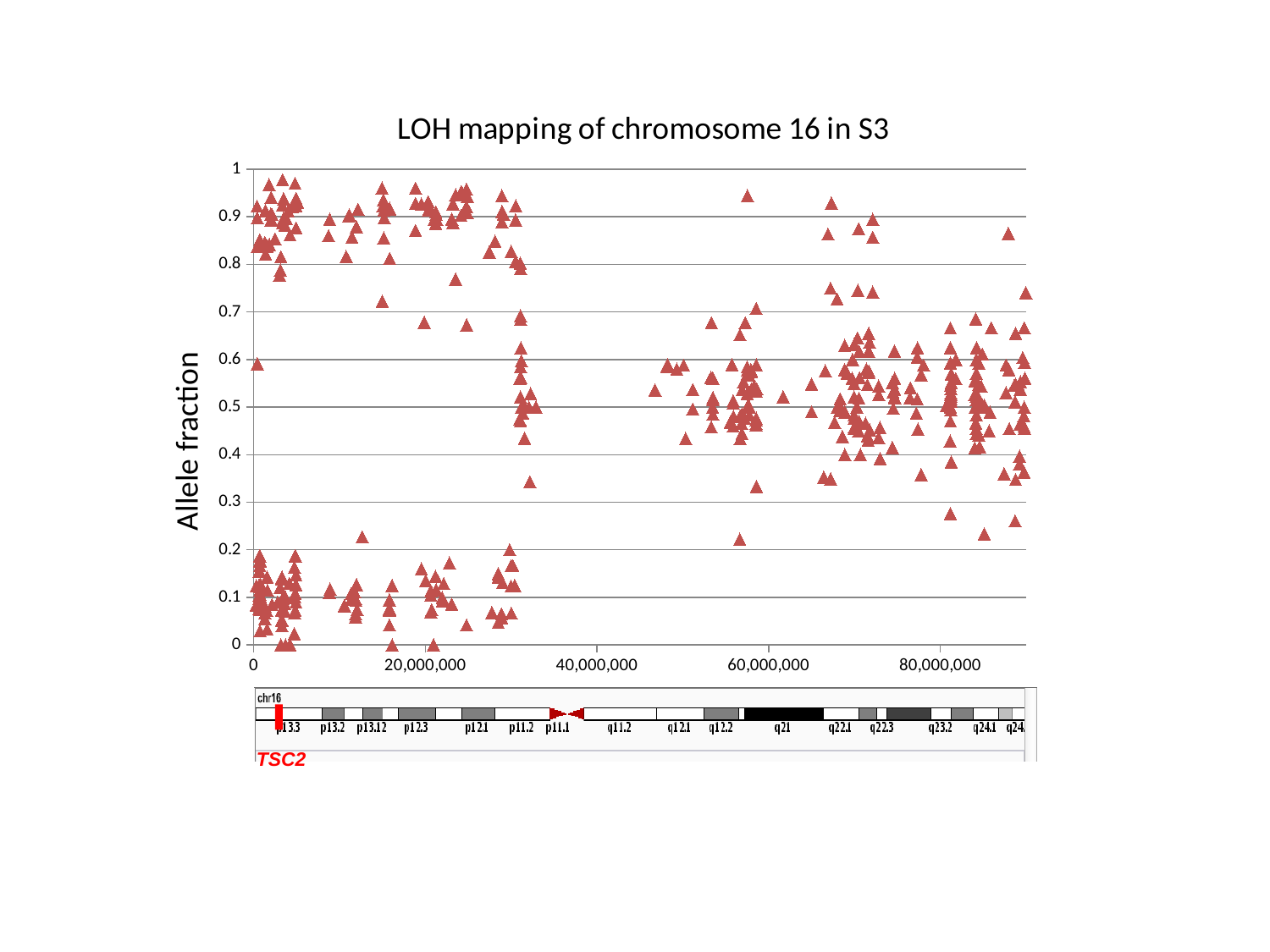

### Chart: LOH mapping of chromosome 16 in S3
| Category | t ratio |
|---|---|Allele fraction
TSC2

## Slide 4
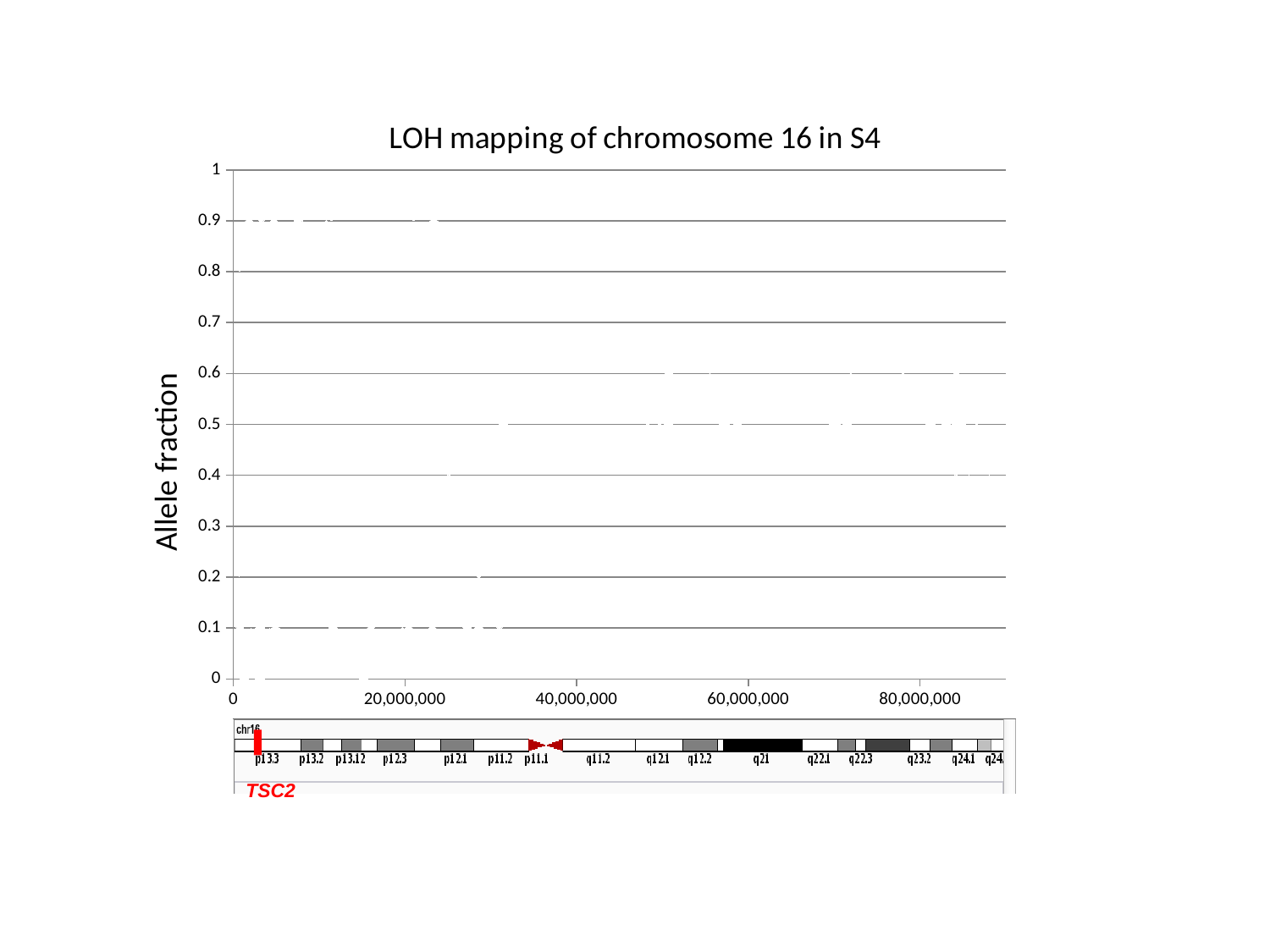

### Chart: LOH mapping of chromosome 16 in S4
| Category | t ratio |
|---|---|Allele fraction
TSC2

## Slide 5
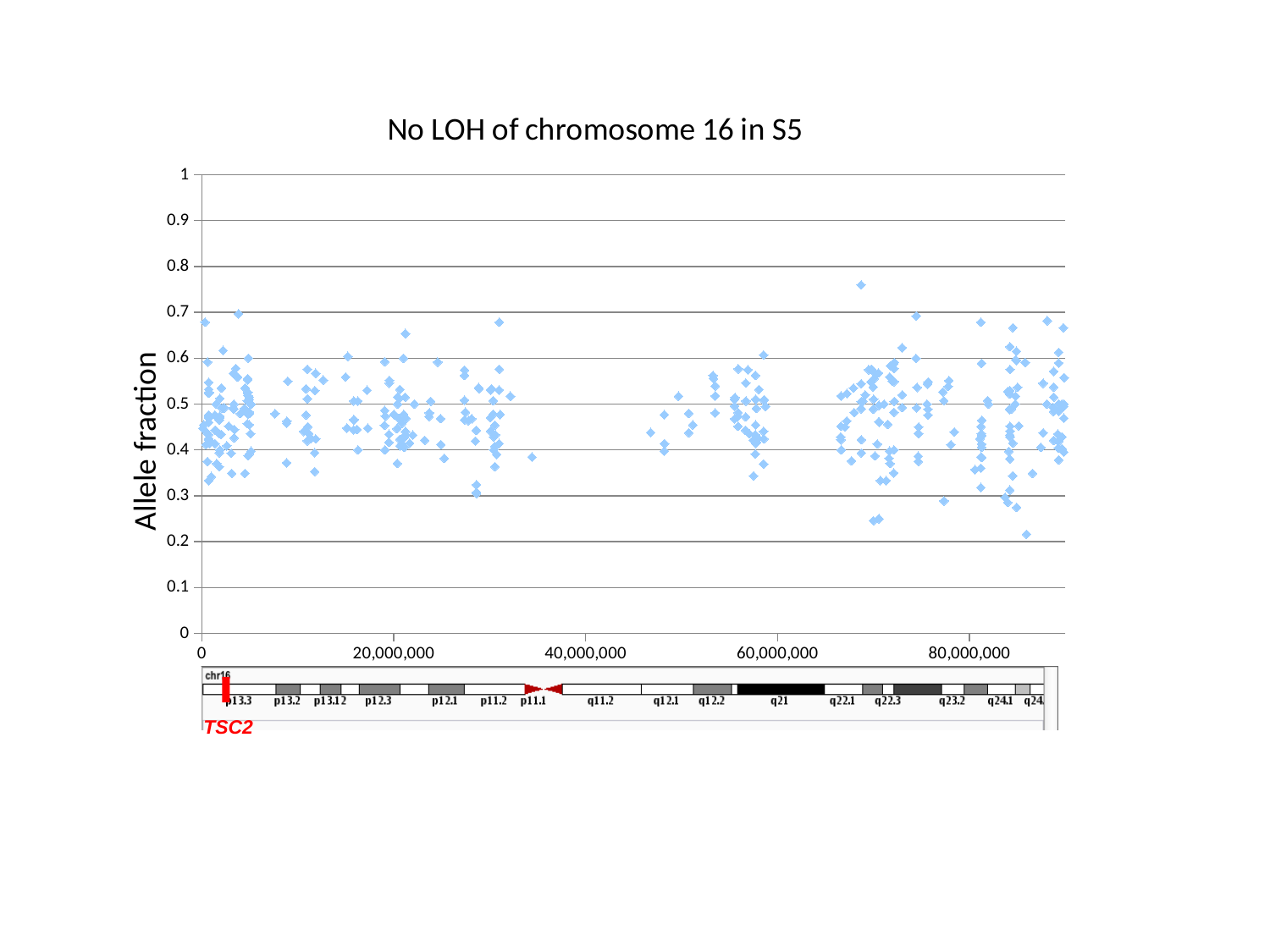

### Chart: No LOH of chromosome 16 in S5
| Category | t_ratio |
|---|---|Allele fraction
TSC2

## Slide 6
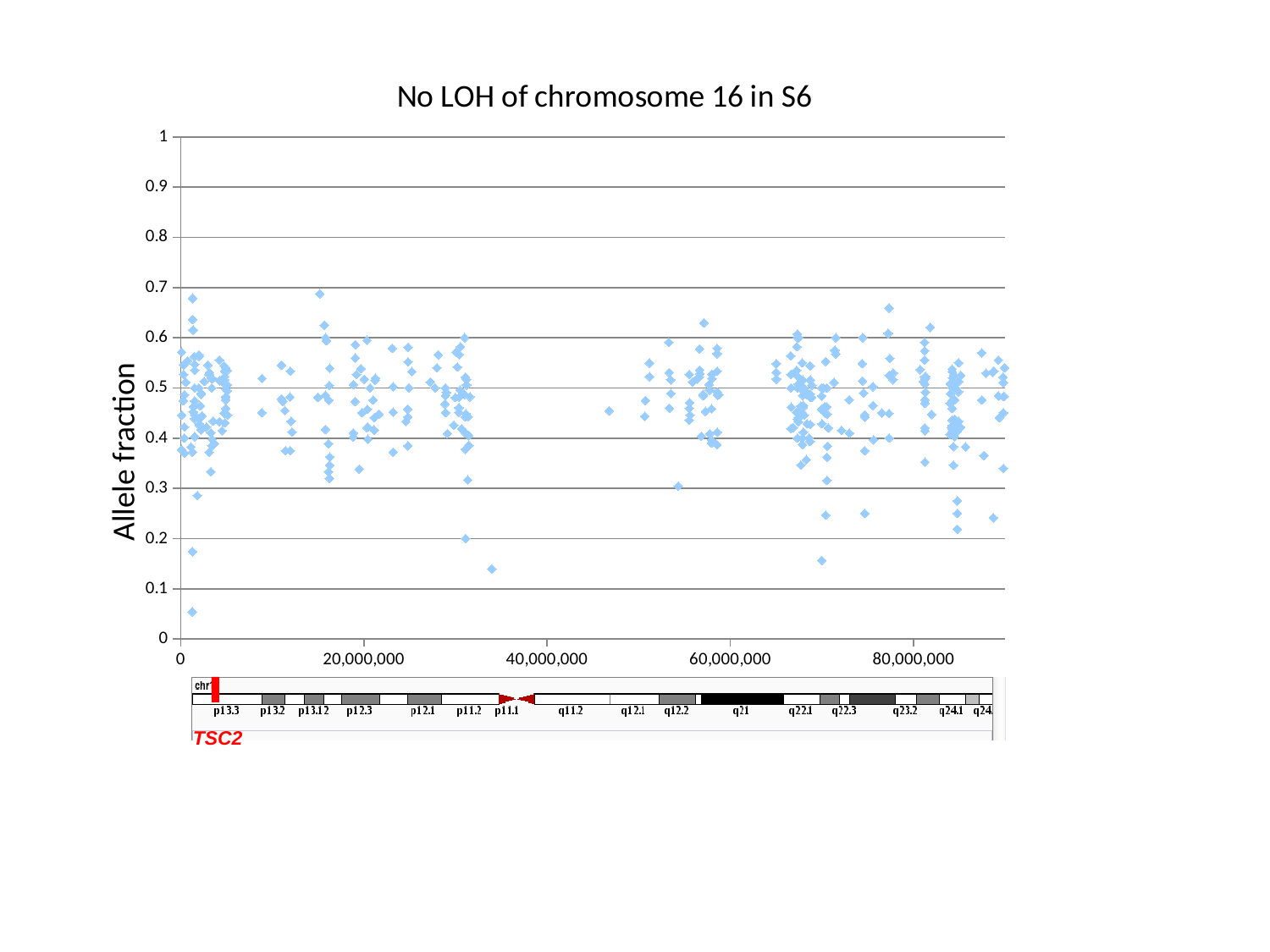

### Chart: No LOH of chromosome 16 in S6
| Category | t_ratio |
|---|---|Allele fraction
TSC2

## Slide 7
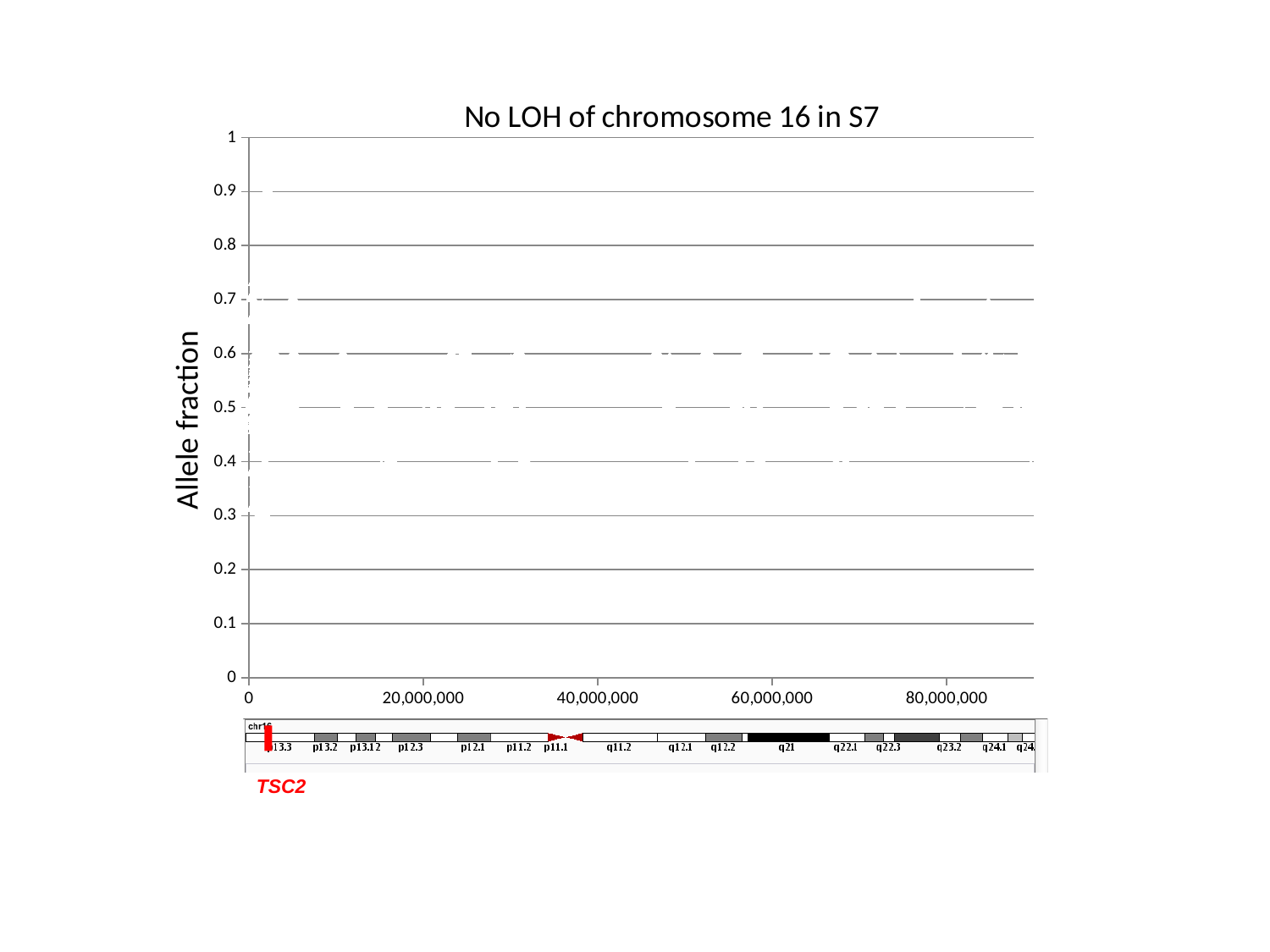

### Chart: No LOH of chromosome 16 in S7
| Category | t ratio |
|---|---|Allele fraction
TSC2

## Slide 8
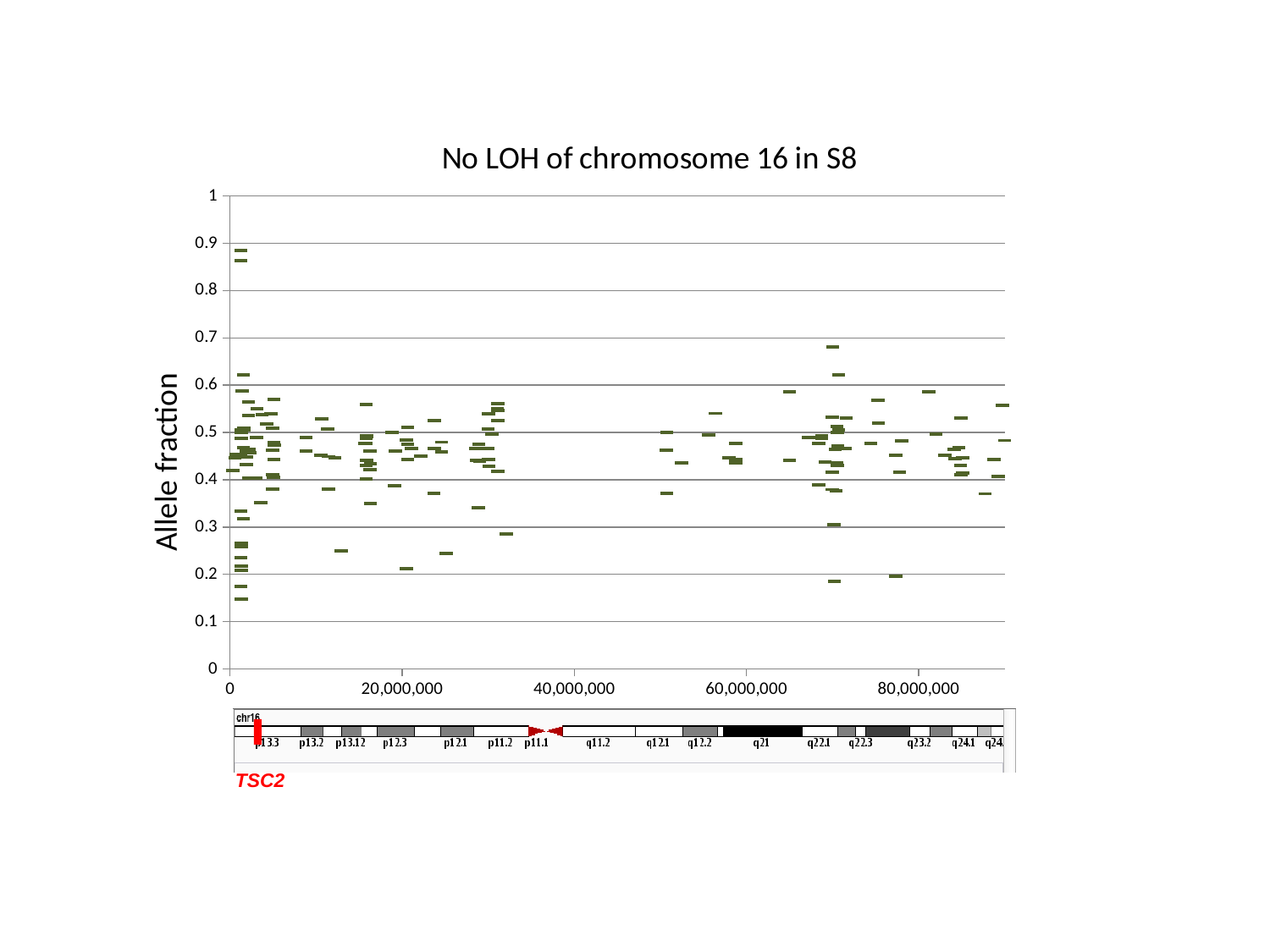

### Chart: No LOH of chromosome 16 in S8
| Category | t_ratio |
|---|---|Allele fraction
TSC2

## Slide 9
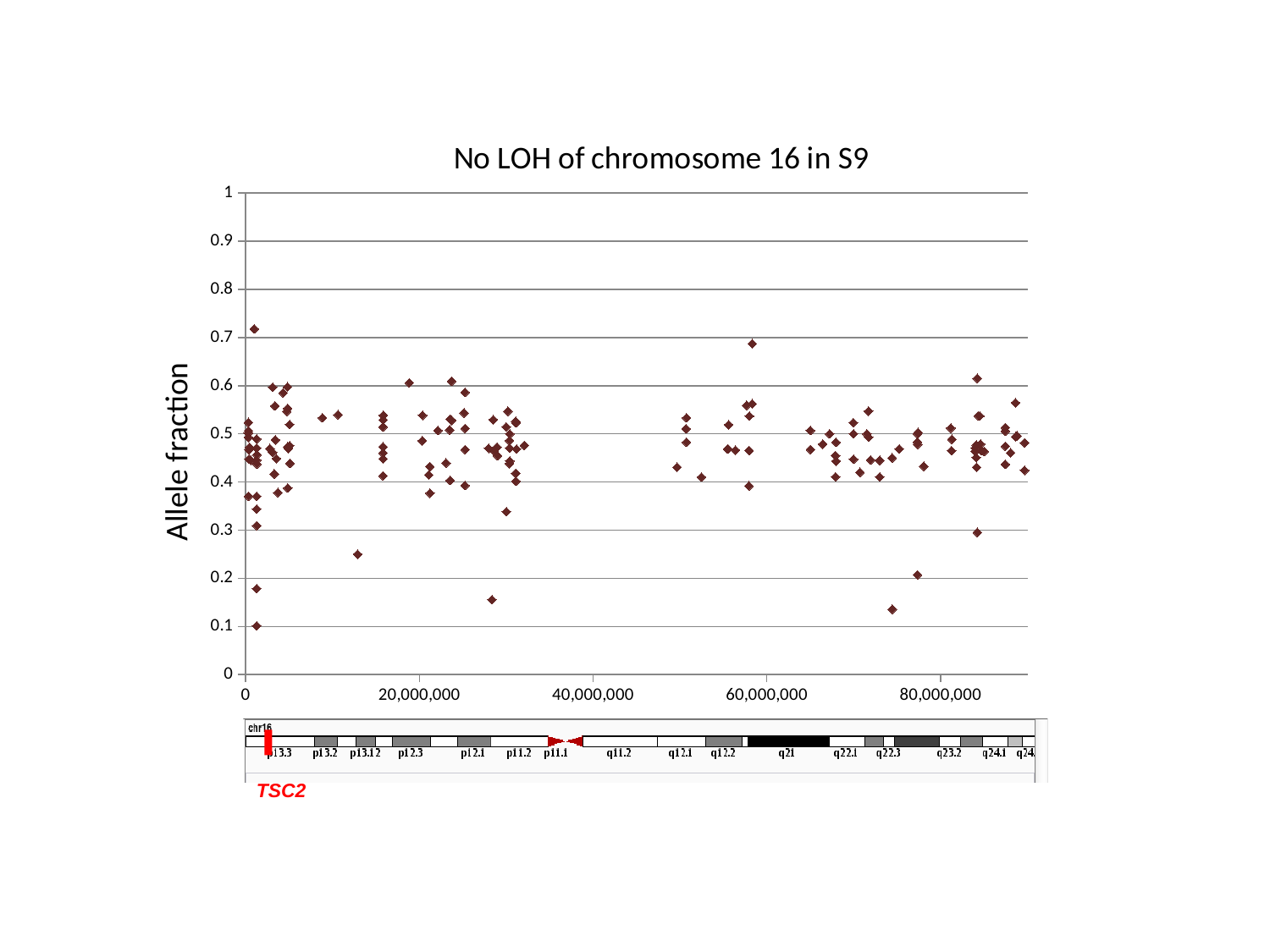

### Chart: No LOH of chromosome 16 in S9
| Category | t_ratio |
|---|---|Allele fraction
TSC2

## Slide 10
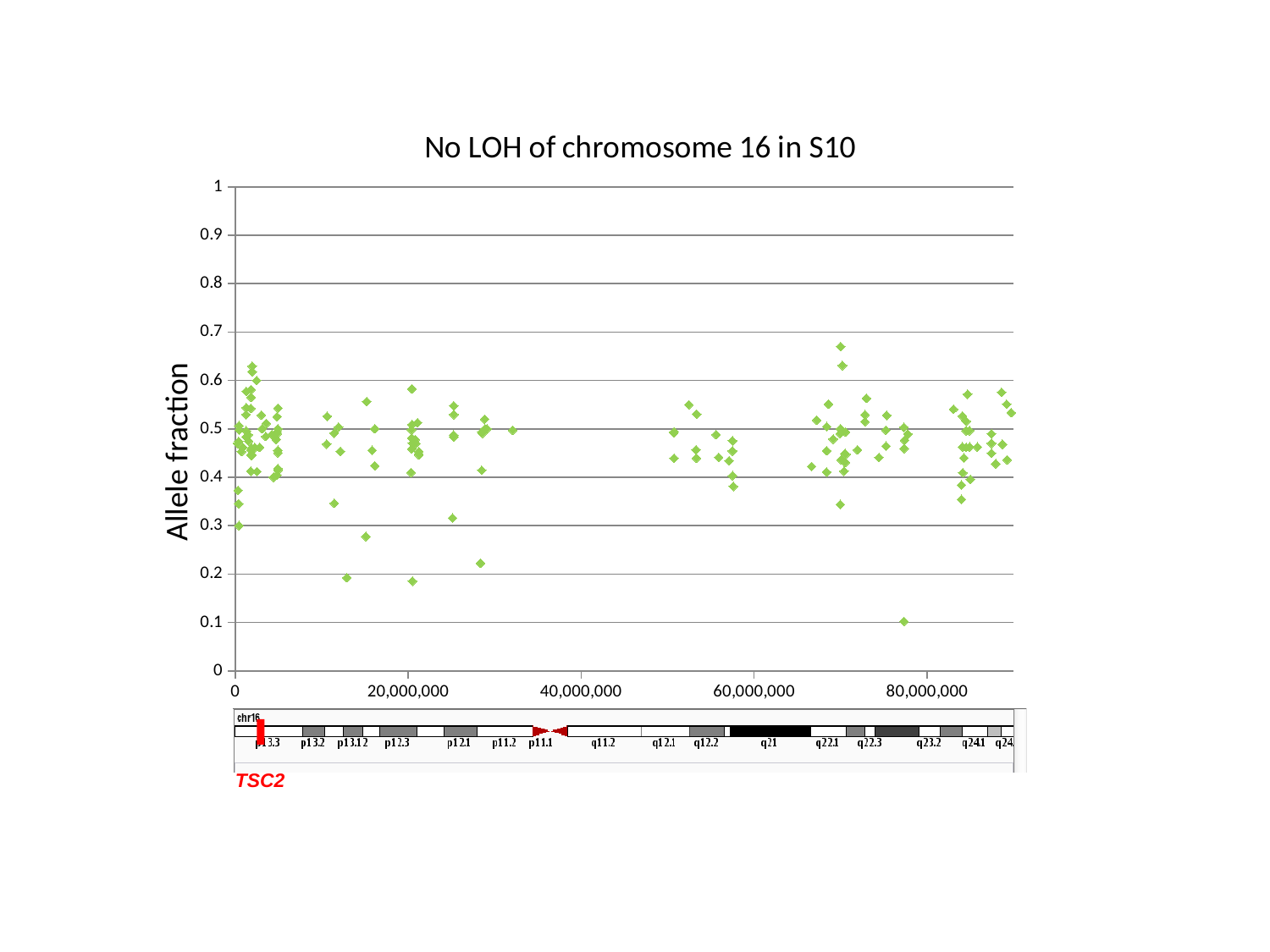

### Chart: No LOH of chromosome 16 in S10
| Category | t_ratio |
|---|---|Allele fraction
TSC2

## Slide 11
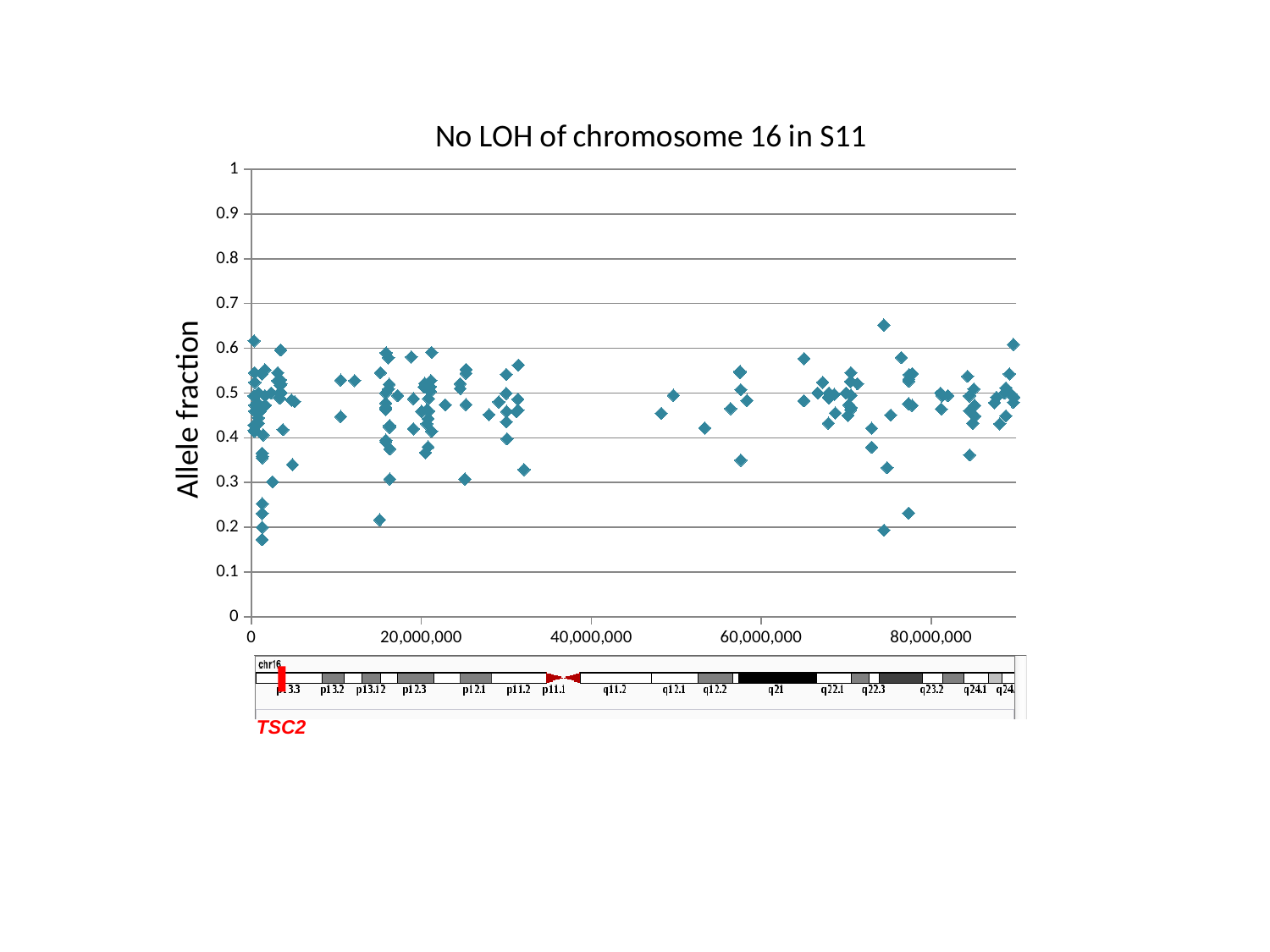

### Chart: No LOH of chromosome 16 in S11
| Category | t_ratio |
|---|---|Allele fraction
TSC2

## Slide 12
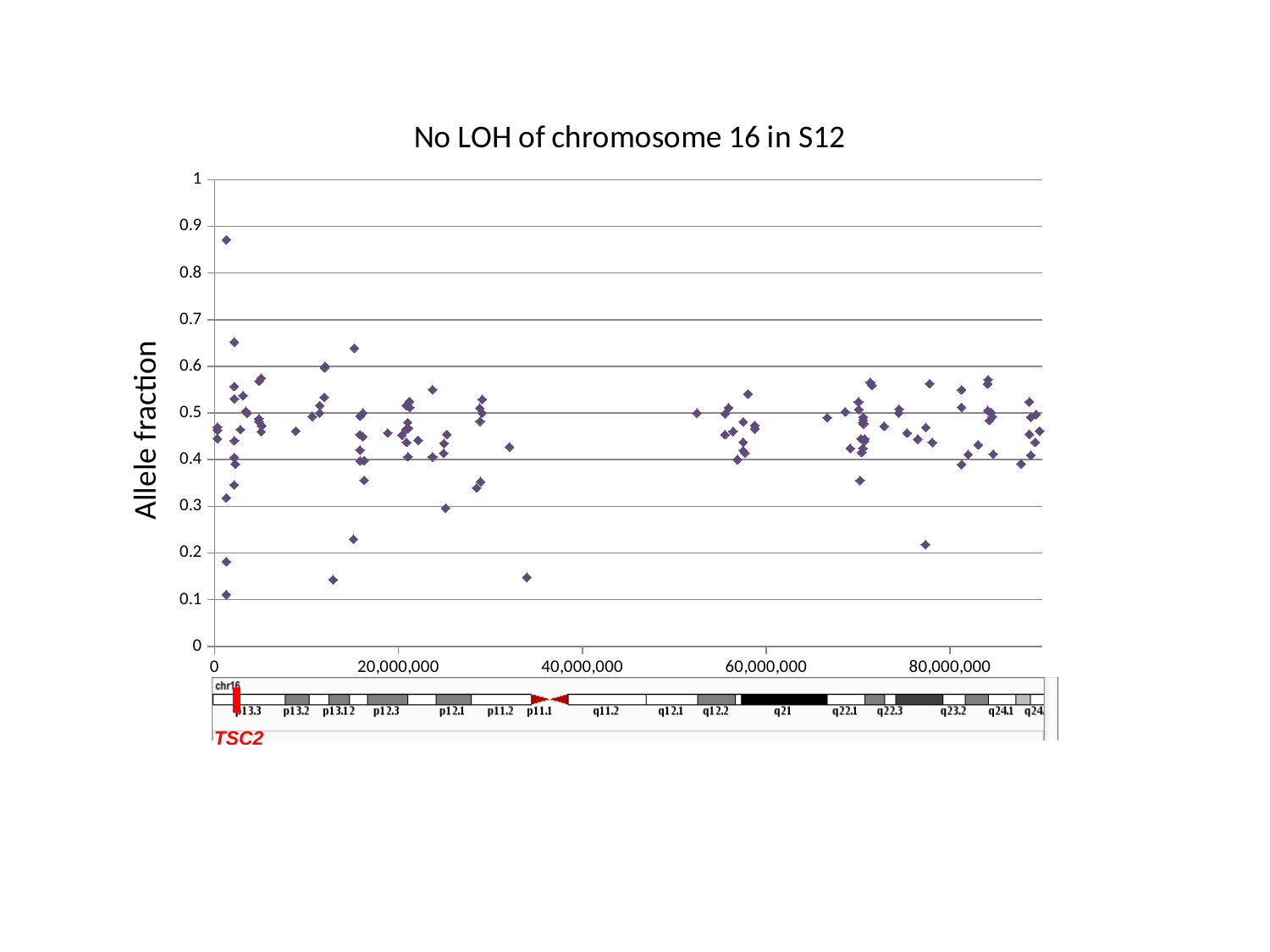

### Chart: No LOH of chromosome 16 in S12
| Category | t_ratio |
|---|---|Allele fraction
TSC2

## Slide 13
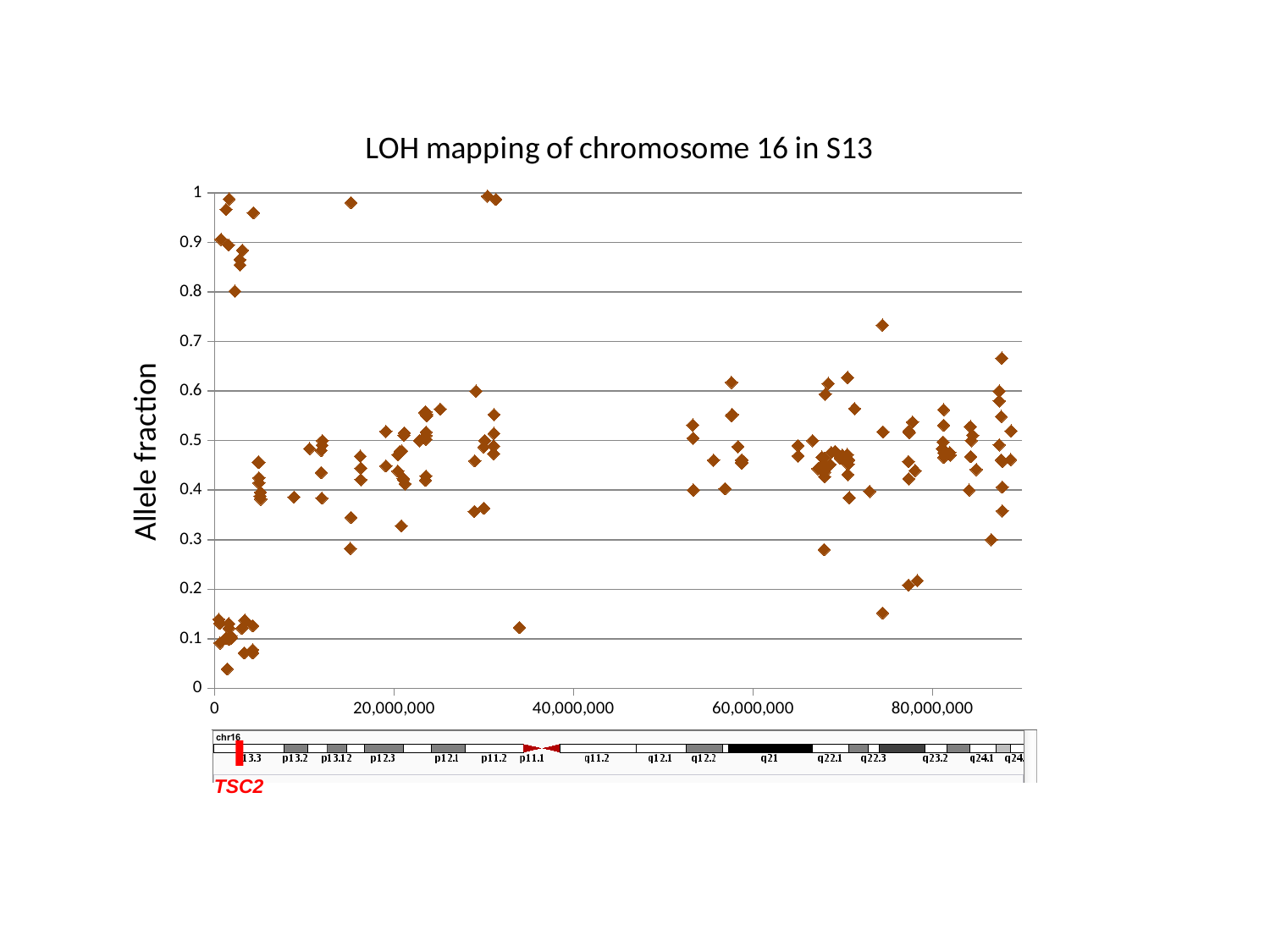

### Chart: LOH mapping of chromosome 16 in S13
| Category | t_ratio |
|---|---|Allele fraction
TSC2

## Slide 14
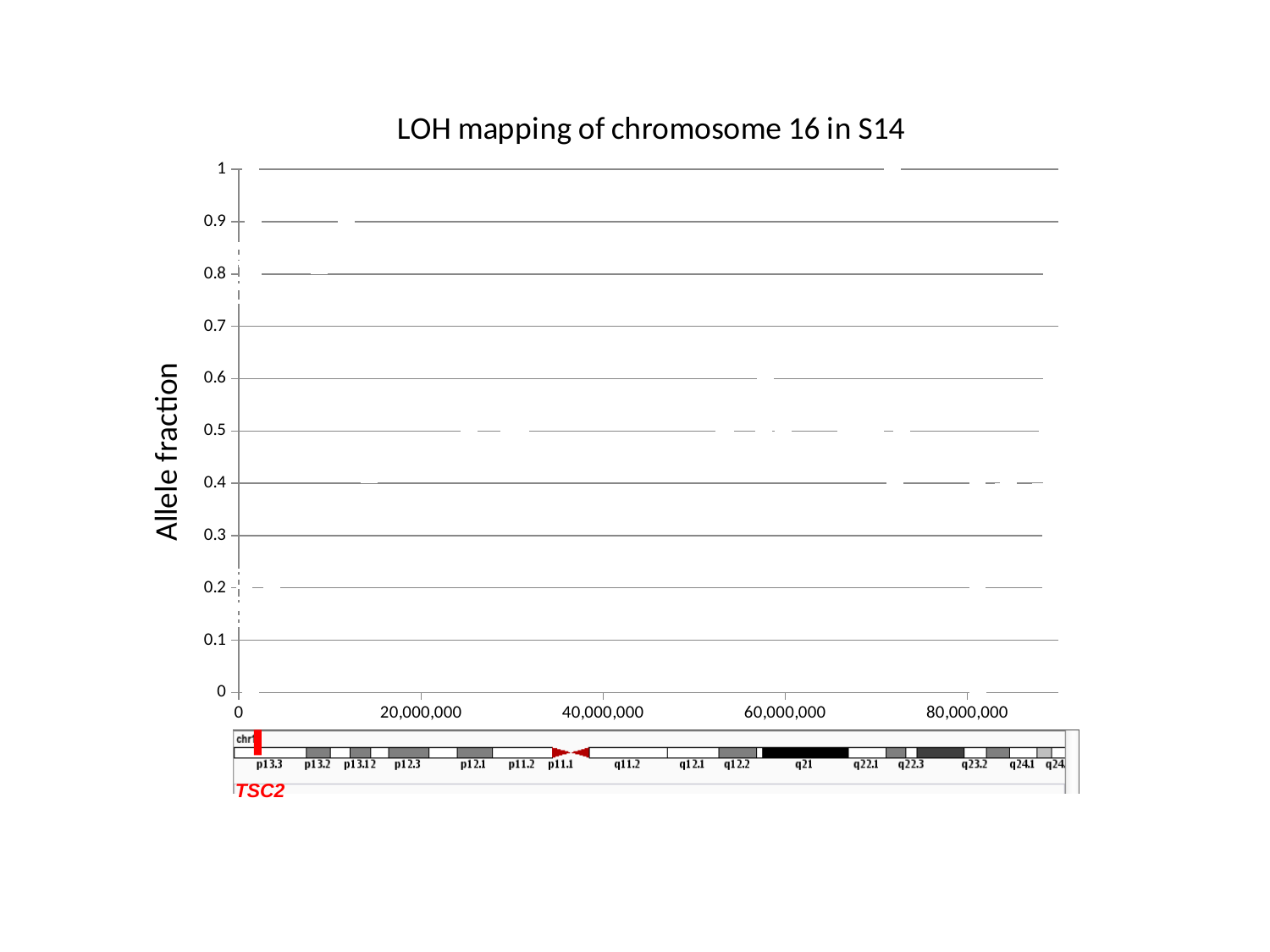

### Chart: LOH mapping of chromosome 16 in S14
| Category | t_ratio T1 |
|---|---|Allele fraction
TSC2

## Slide 15
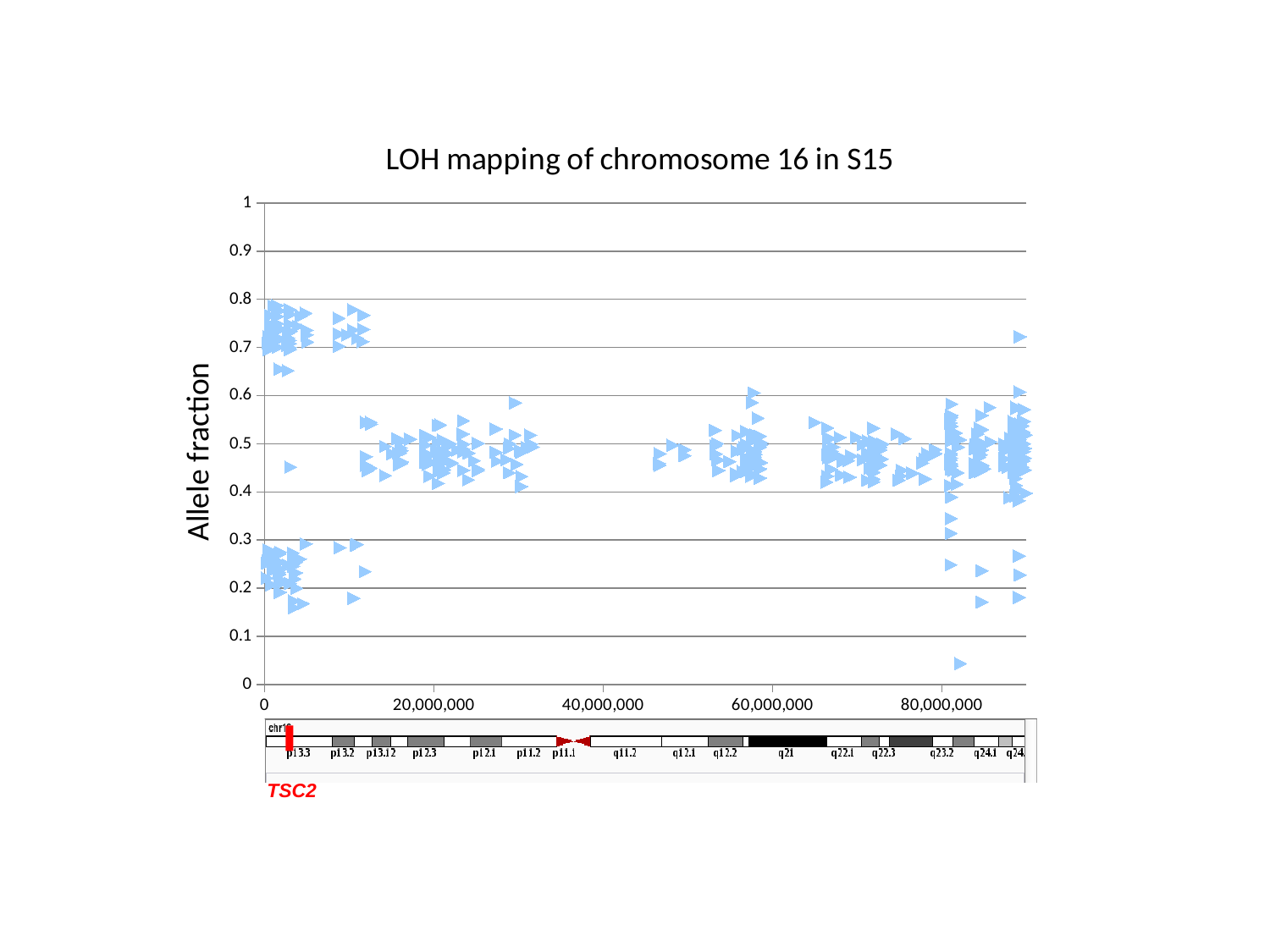

### Chart: LOH mapping of chromosome 16 in S15
| Category | t_ratio |
|---|---|Allele fraction
TSC2

## Slide 16
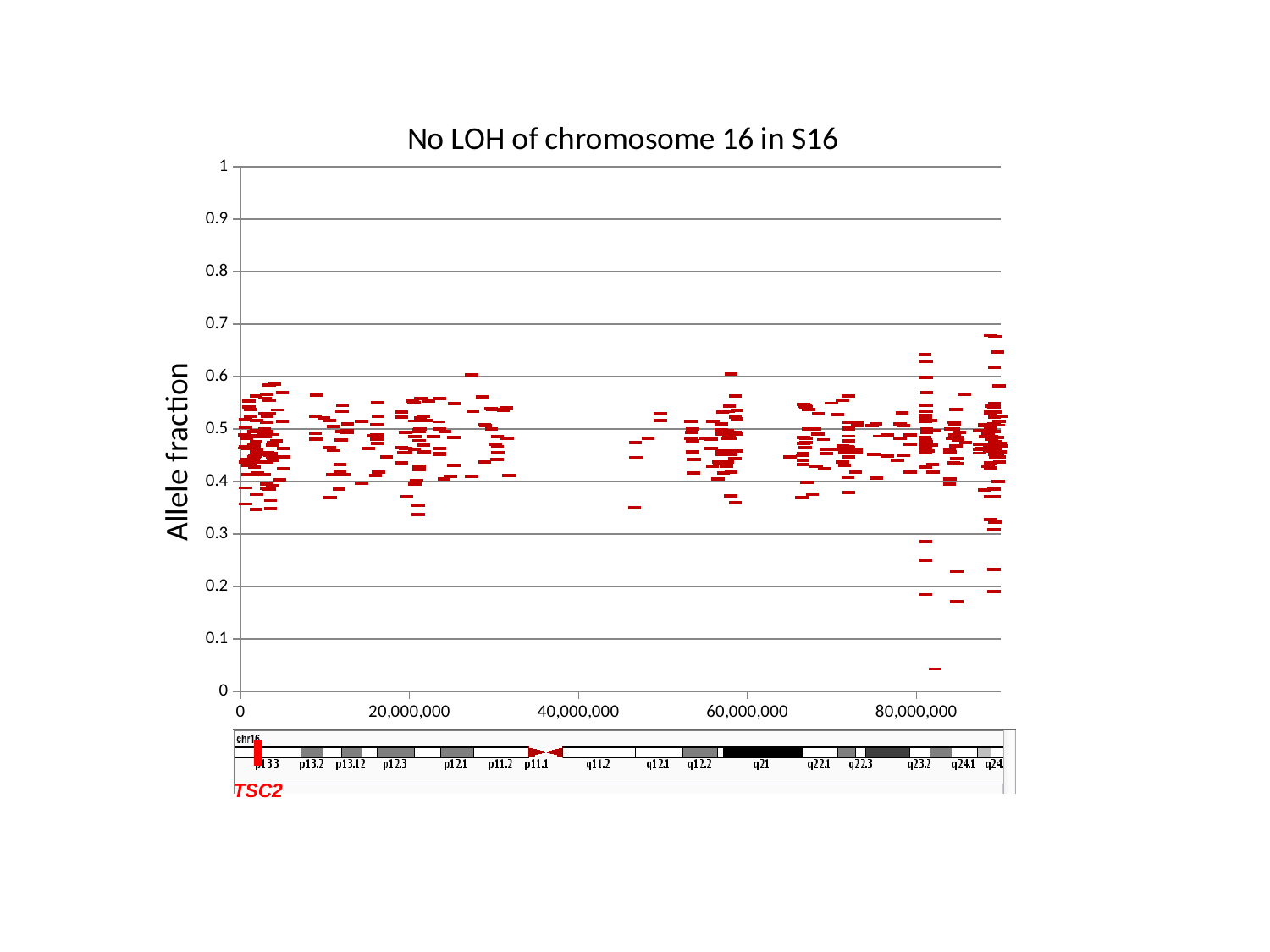

### Chart: No LOH of chromosome 16 in S16
| Category | |
|---|---|Allele fraction
TSC2

## Slide 17
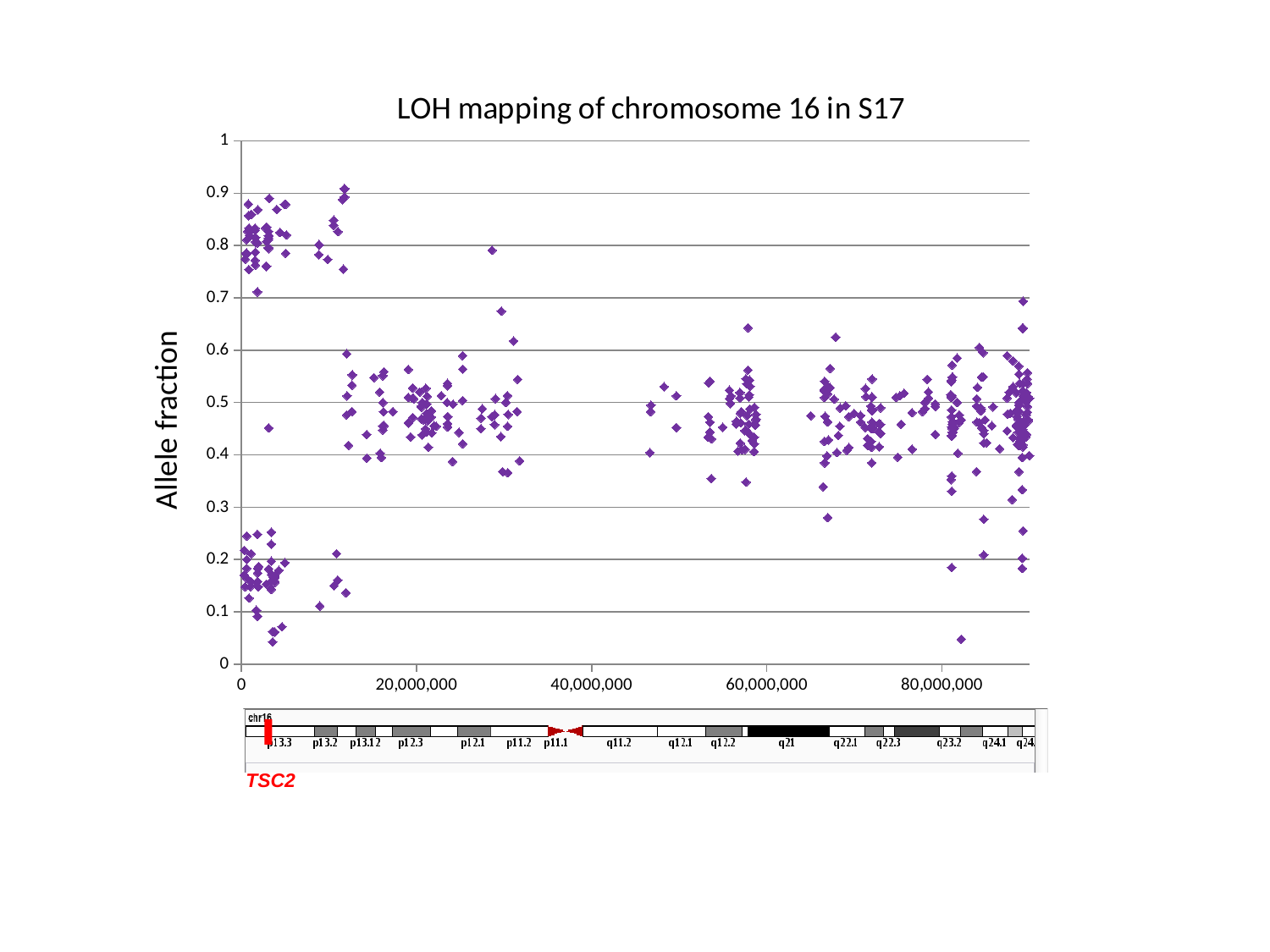

### Chart: LOH mapping of chromosome 16 in S17
| Category | |
|---|---|Allele fraction
TSC2

## Slide 18
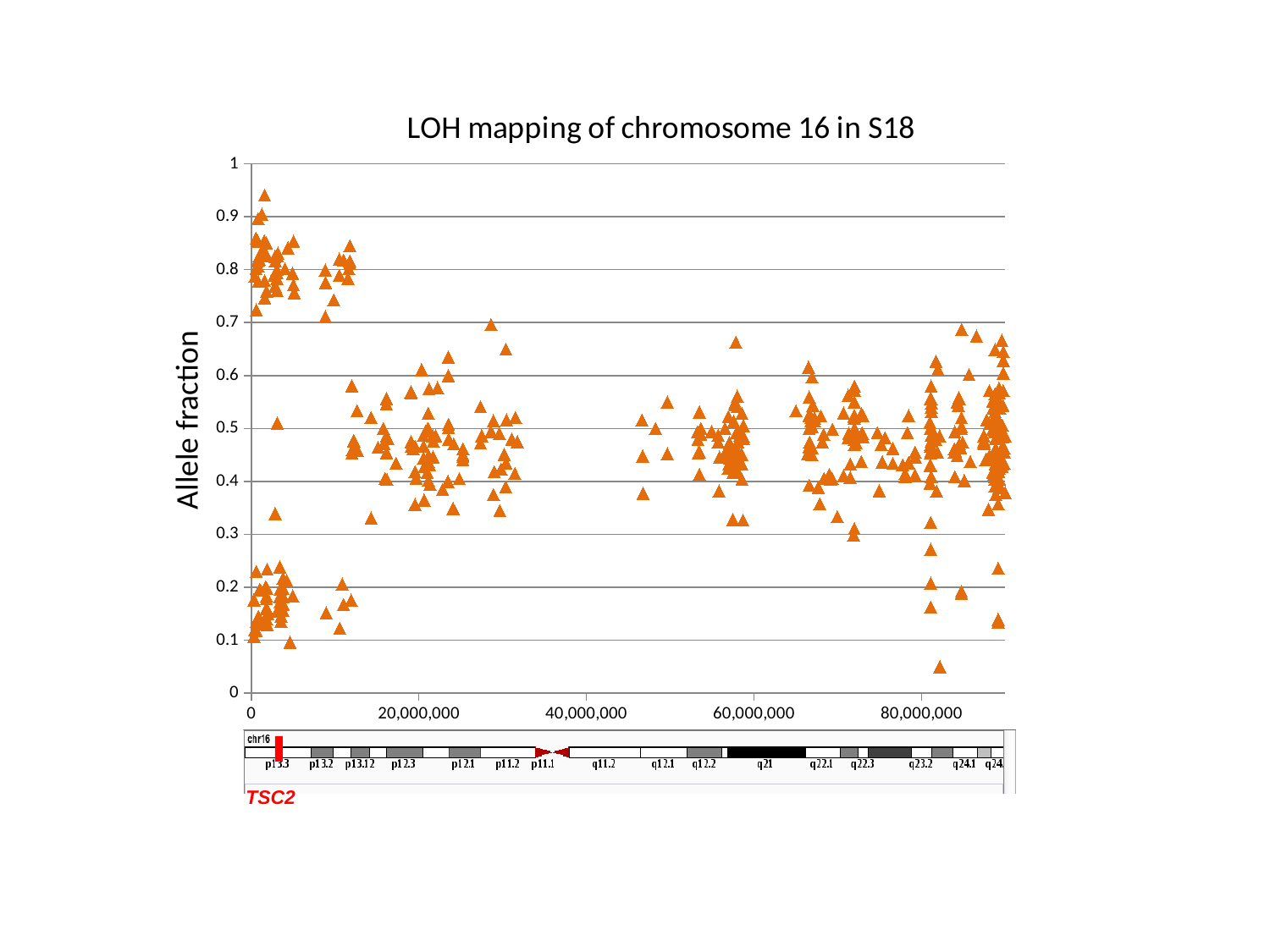

### Chart: LOH mapping of chromosome 16 in S18
| Category | |
|---|---|Allele fraction
TSC2

## Slide 19
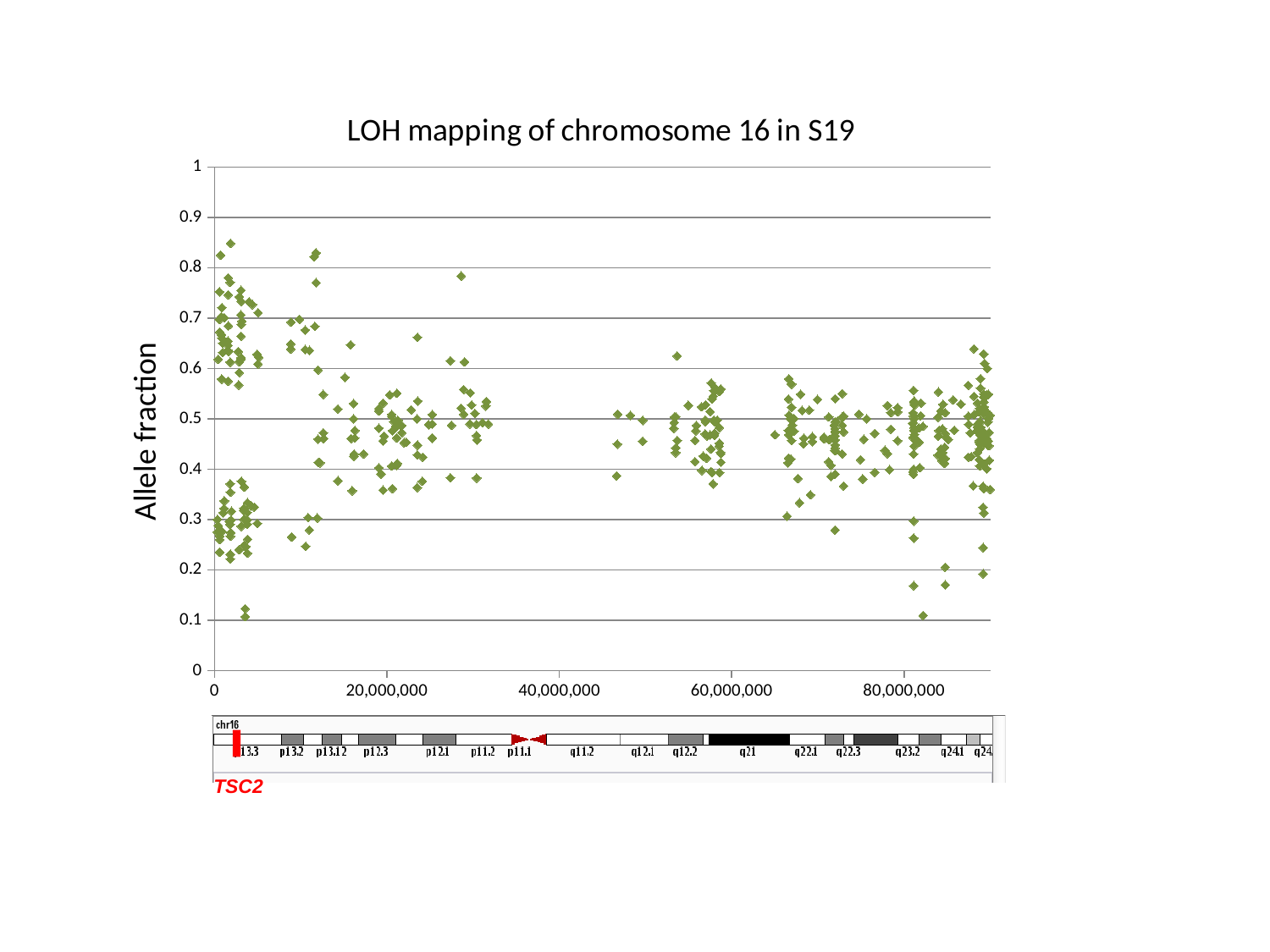

### Chart: LOH mapping of chromosome 16 in S19
| Category | |
|---|---|Allele fraction
TSC2

## Slide 20
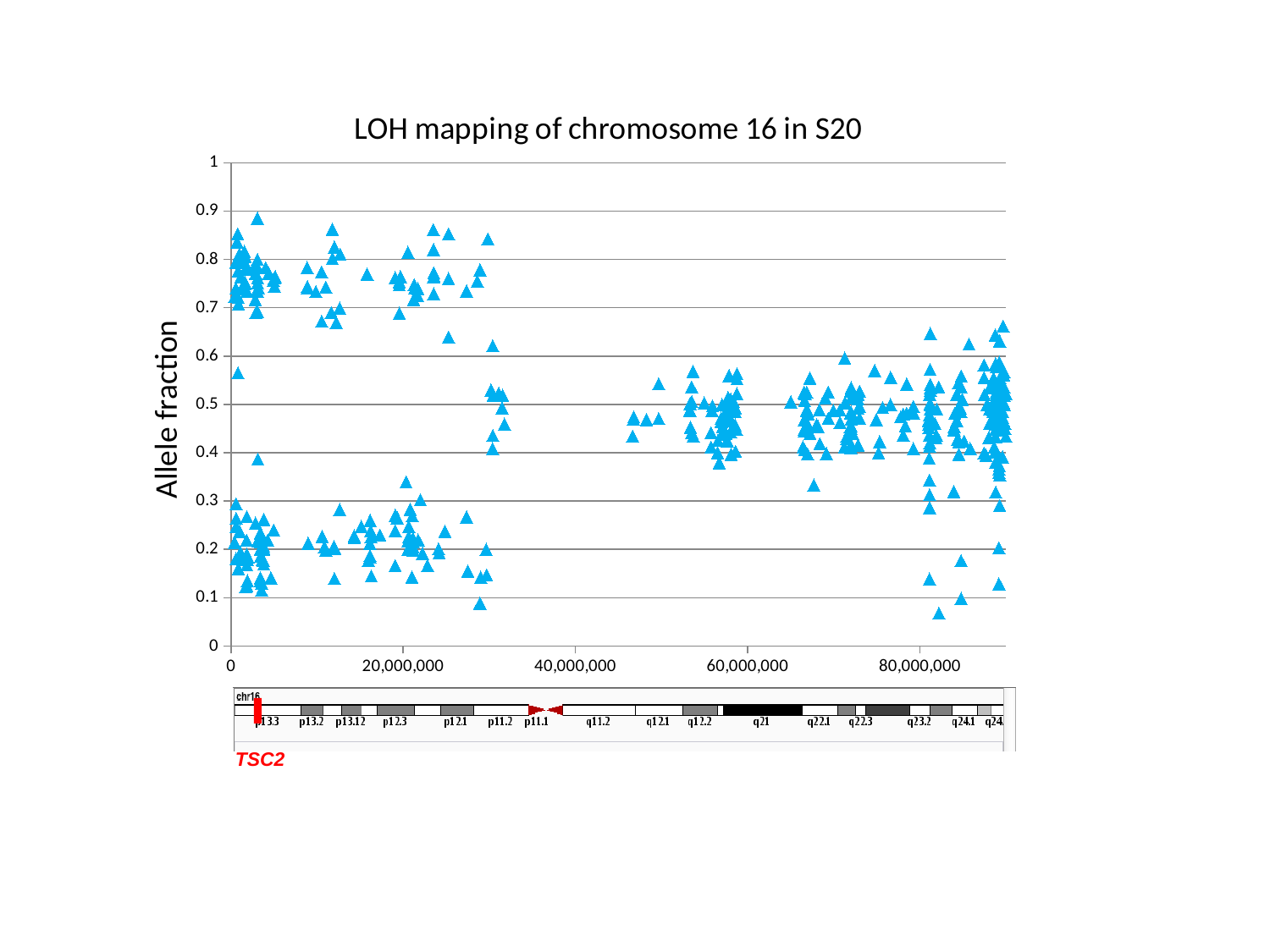

### Chart: LOH mapping of chromosome 16 in S20
| Category | |
|---|---|Allele fraction
TSC2

## Slide 21
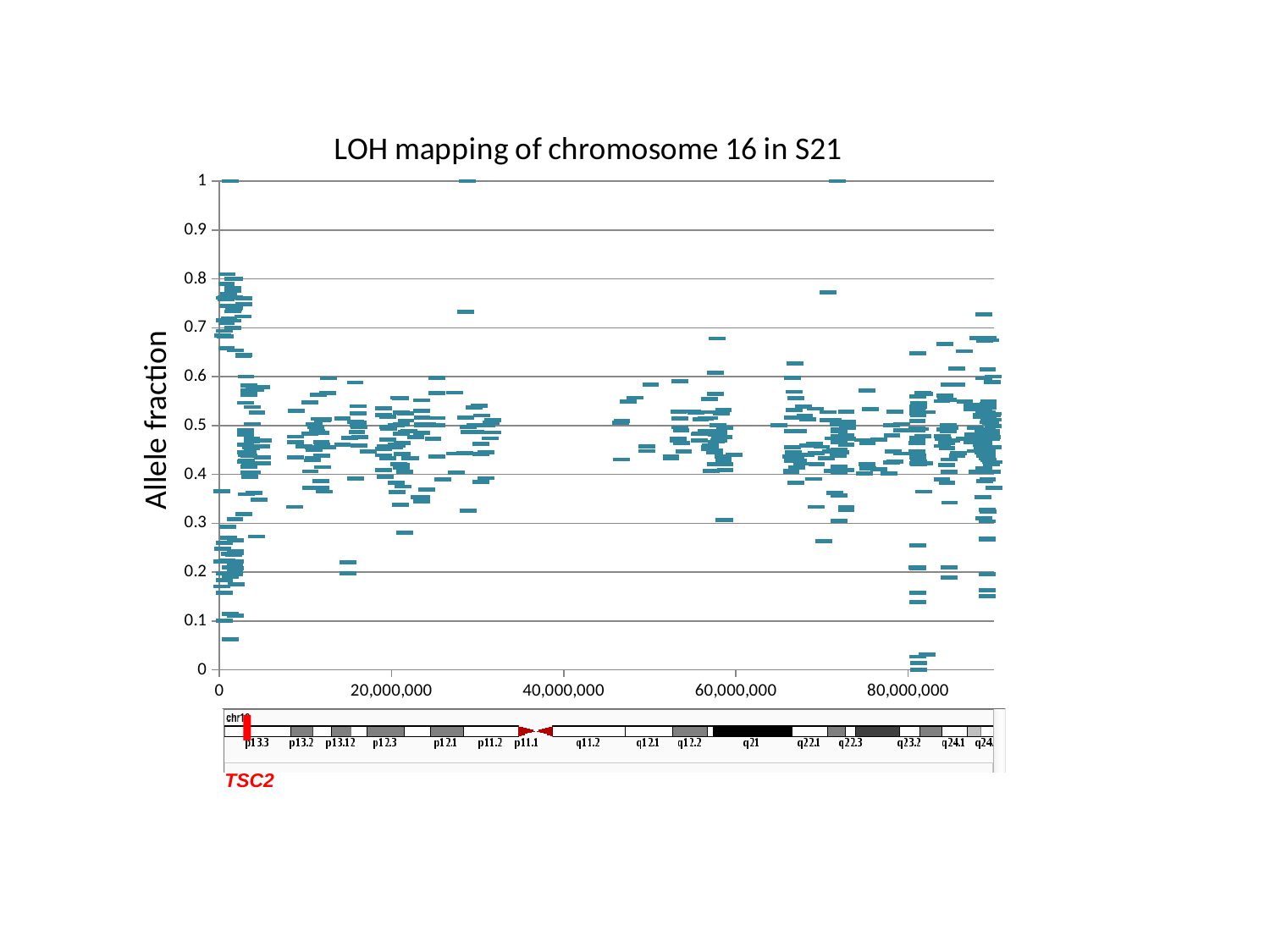

### Chart: LOH mapping of chromosome 16 in S21
| Category | t_ratio T8 |
|---|---|Allele fraction
TSC2

## Slide 22
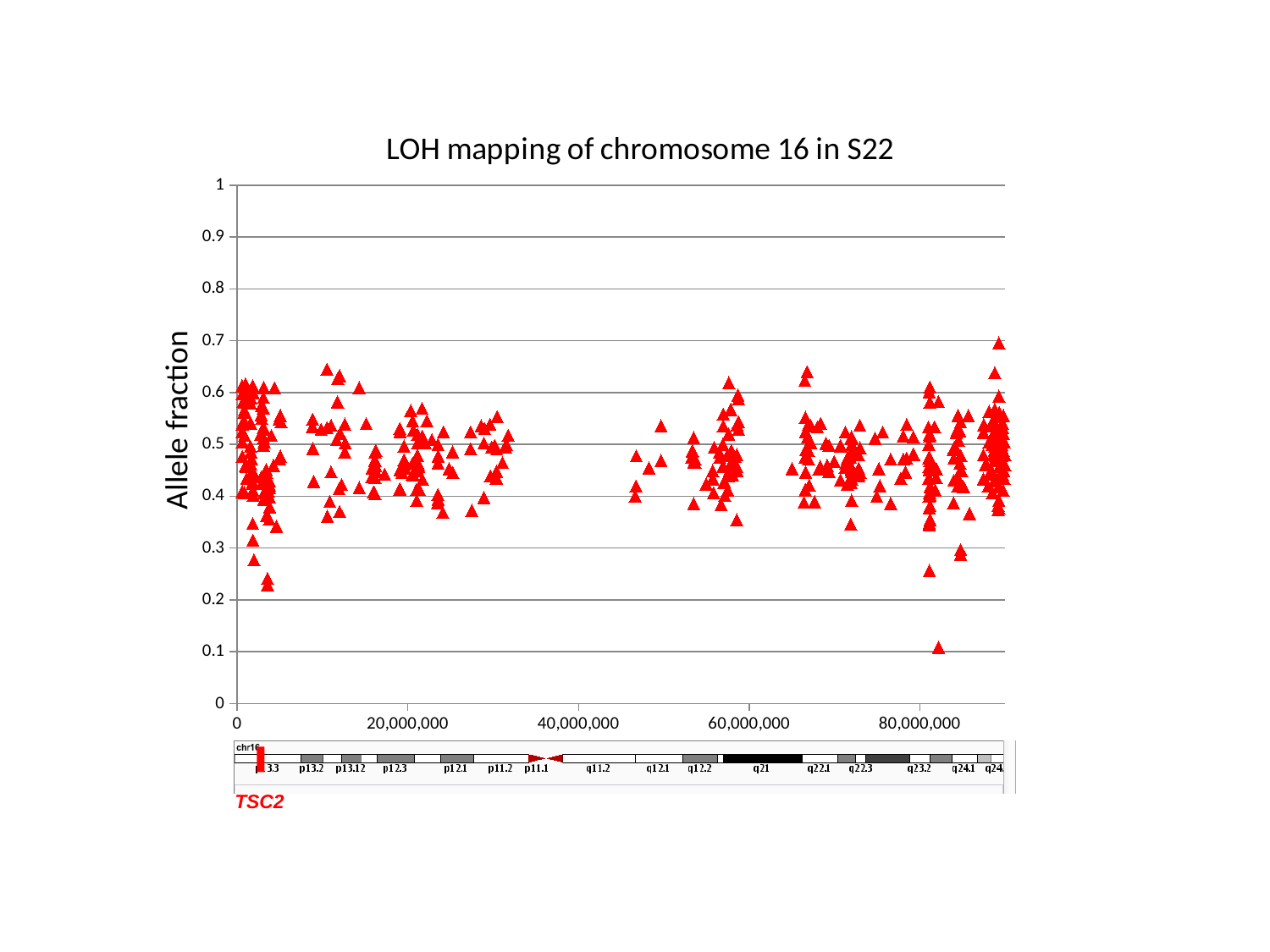

### Chart: LOH mapping of chromosome 16 in S22
| Category | |
|---|---|Allele fraction
TSC2

## Slide 23
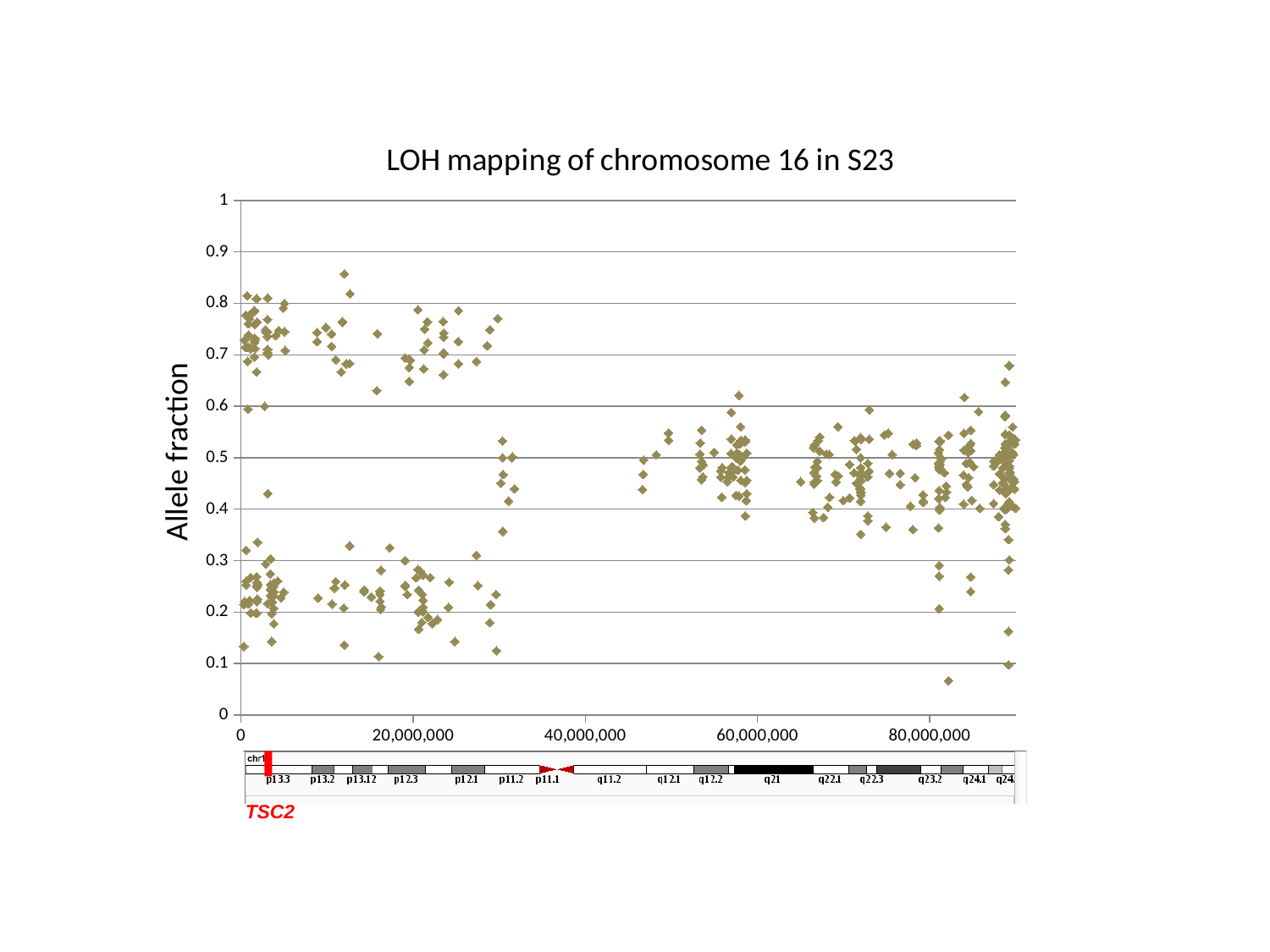

### Chart: LOH mapping of chromosome 16 in S23
| Category | |
|---|---|Allele fraction
TSC2

## Slide 24
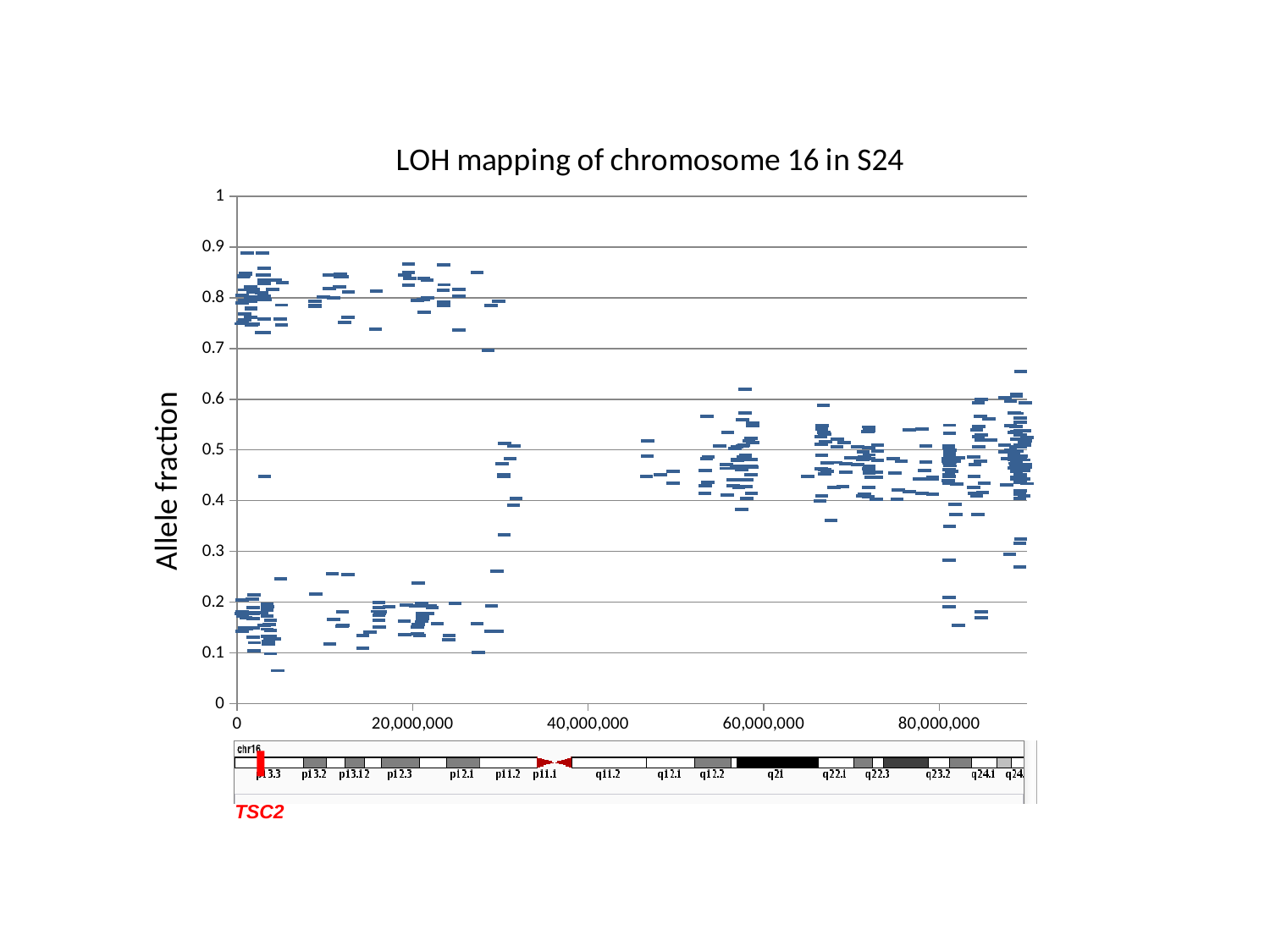

### Chart: LOH mapping of chromosome 16 in S24
| Category | |
|---|---|Allele fraction
TSC2

## Slide 25
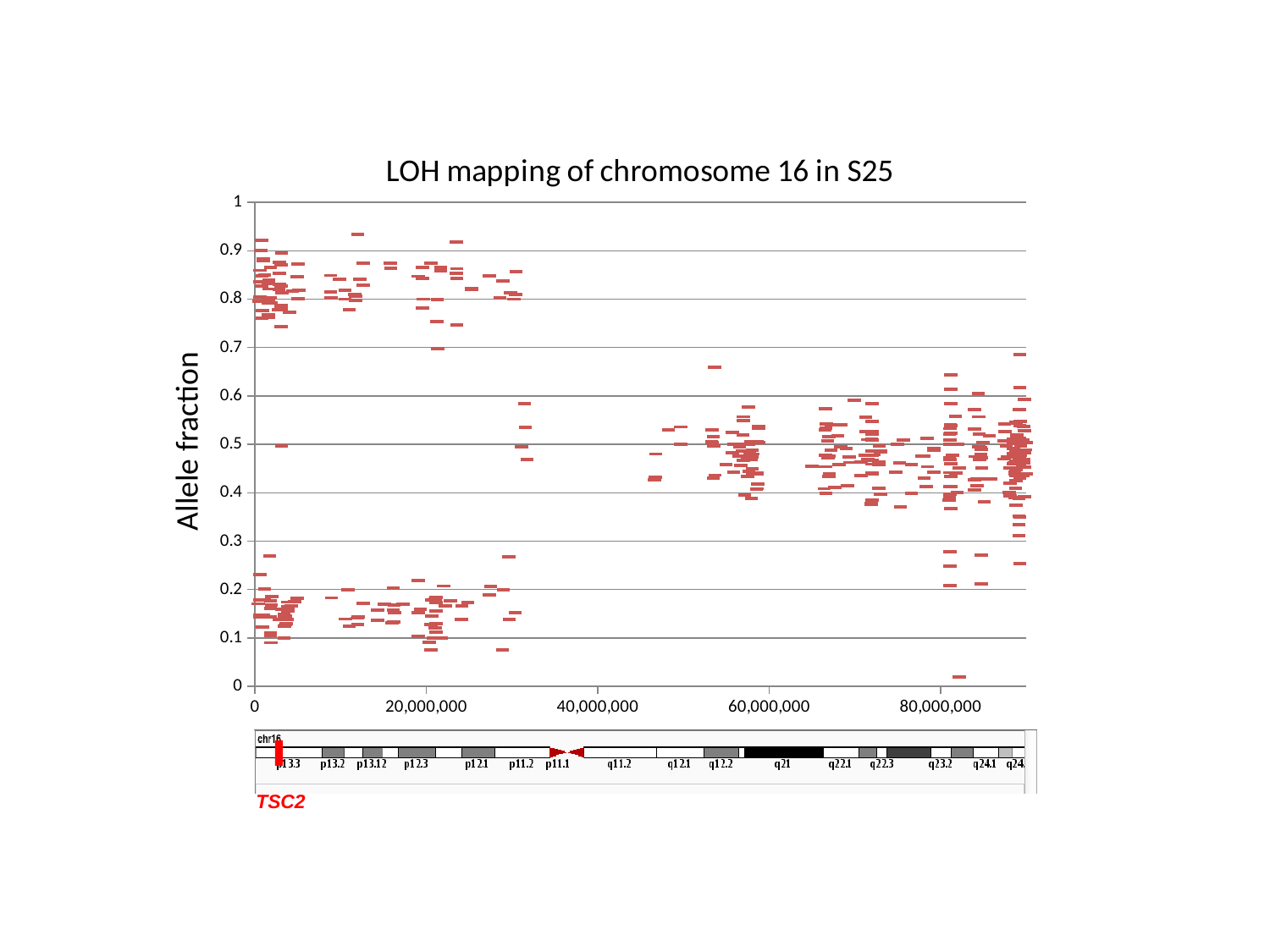

### Chart: LOH mapping of chromosome 16 in S25
| Category | |
|---|---|Allele fraction
TSC2

## Slide 26
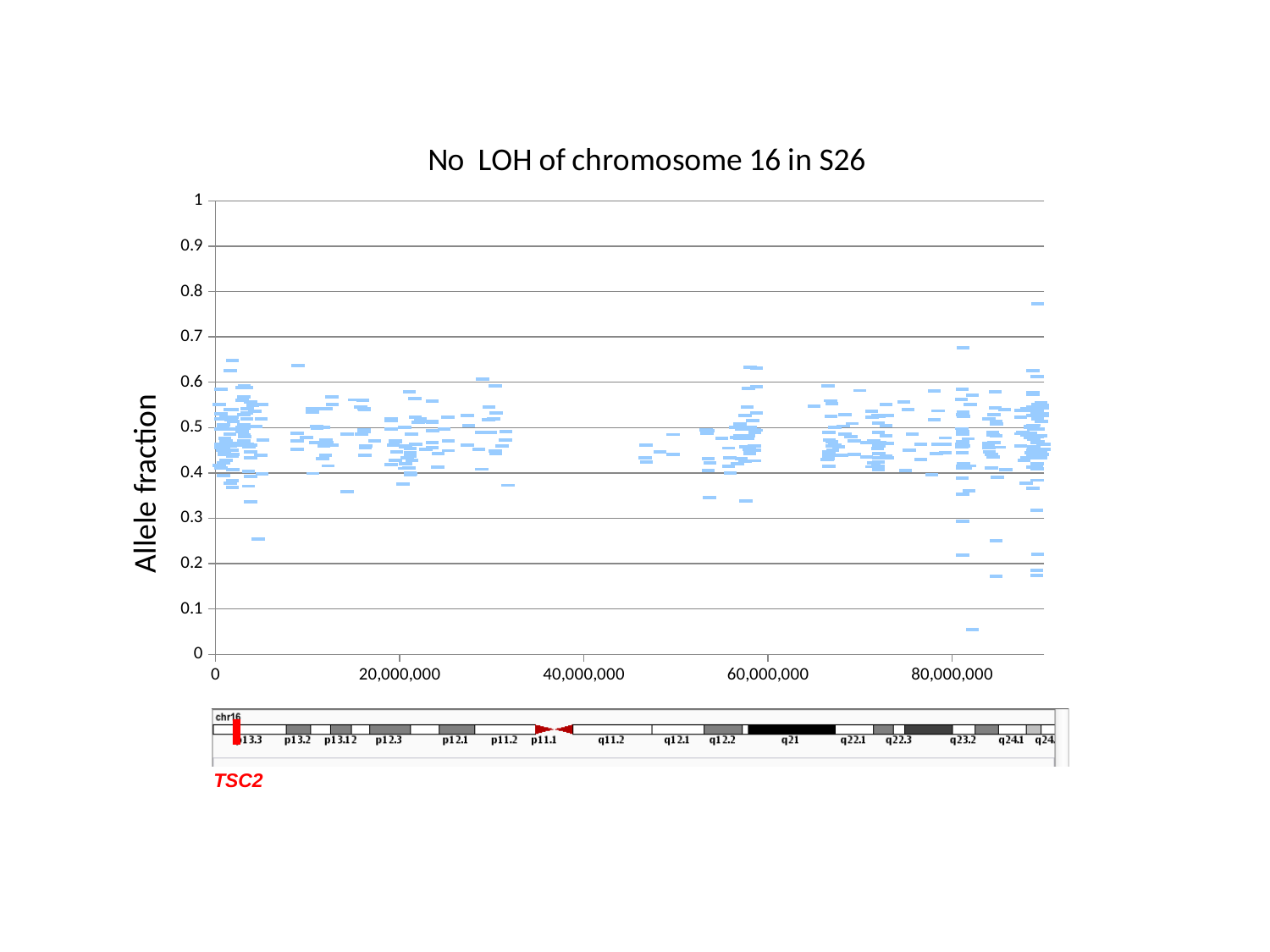

### Chart: No LOH of chromosome 16 in S26
| Category | |
|---|---|Allele fraction
TSC2

## Slide 27
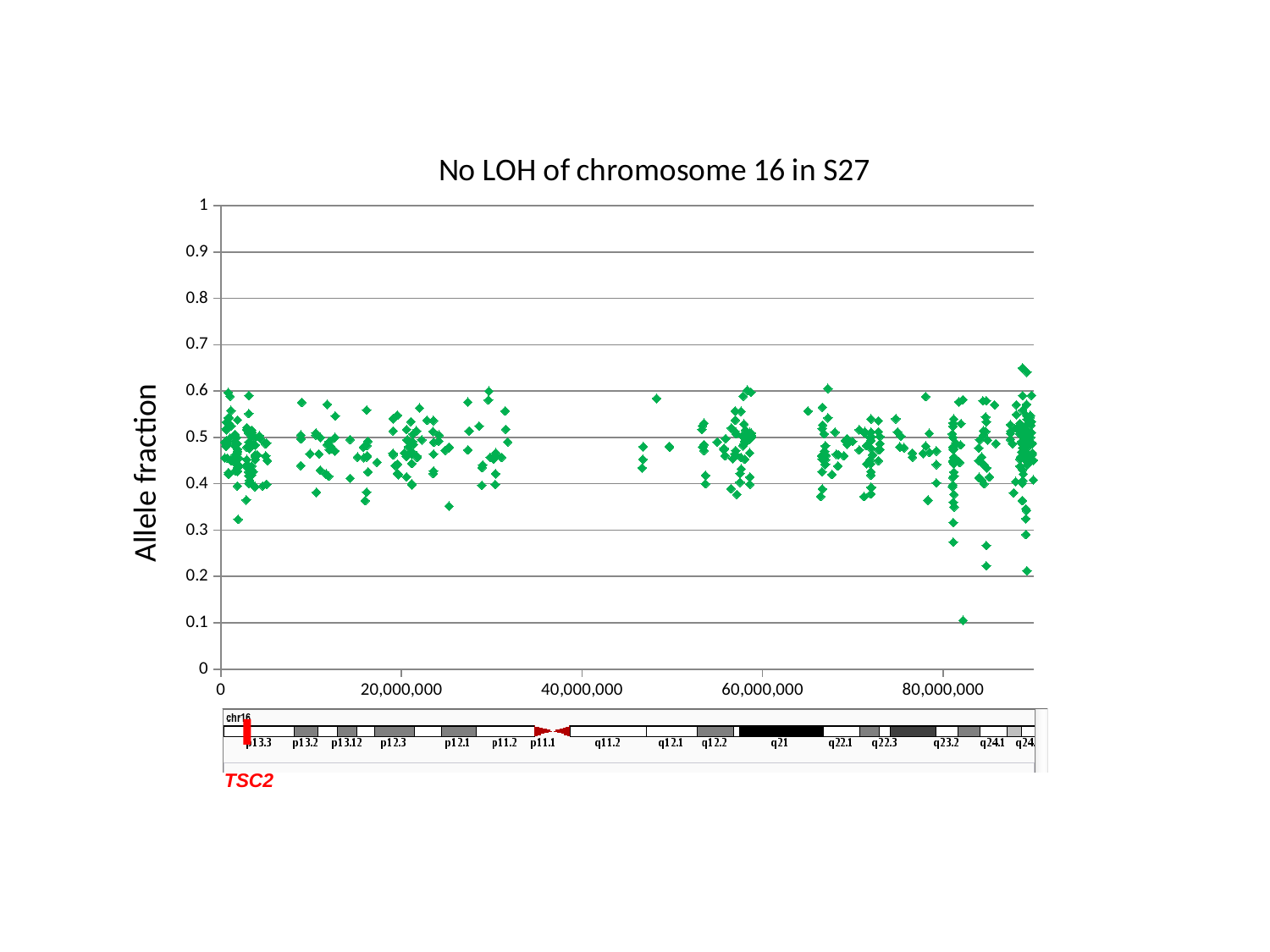

### Chart: No LOH of chromosome 16 in S27
| Category | |
|---|---|Allele fraction
TSC2

## Slide 28
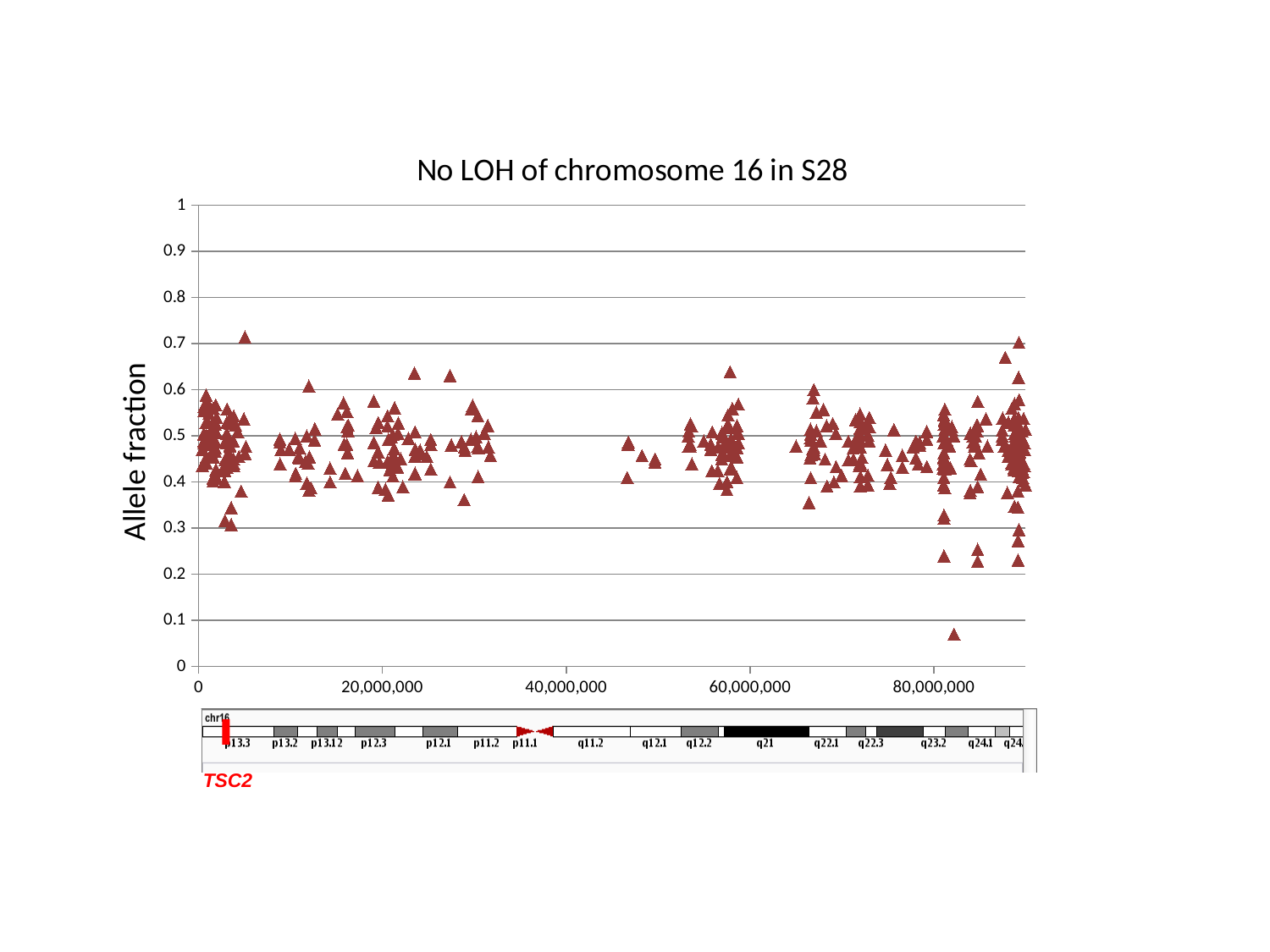

### Chart: No LOH of chromosome 16 in S28
| Category | |
|---|---|Allele fraction
TSC2

## Slide 29
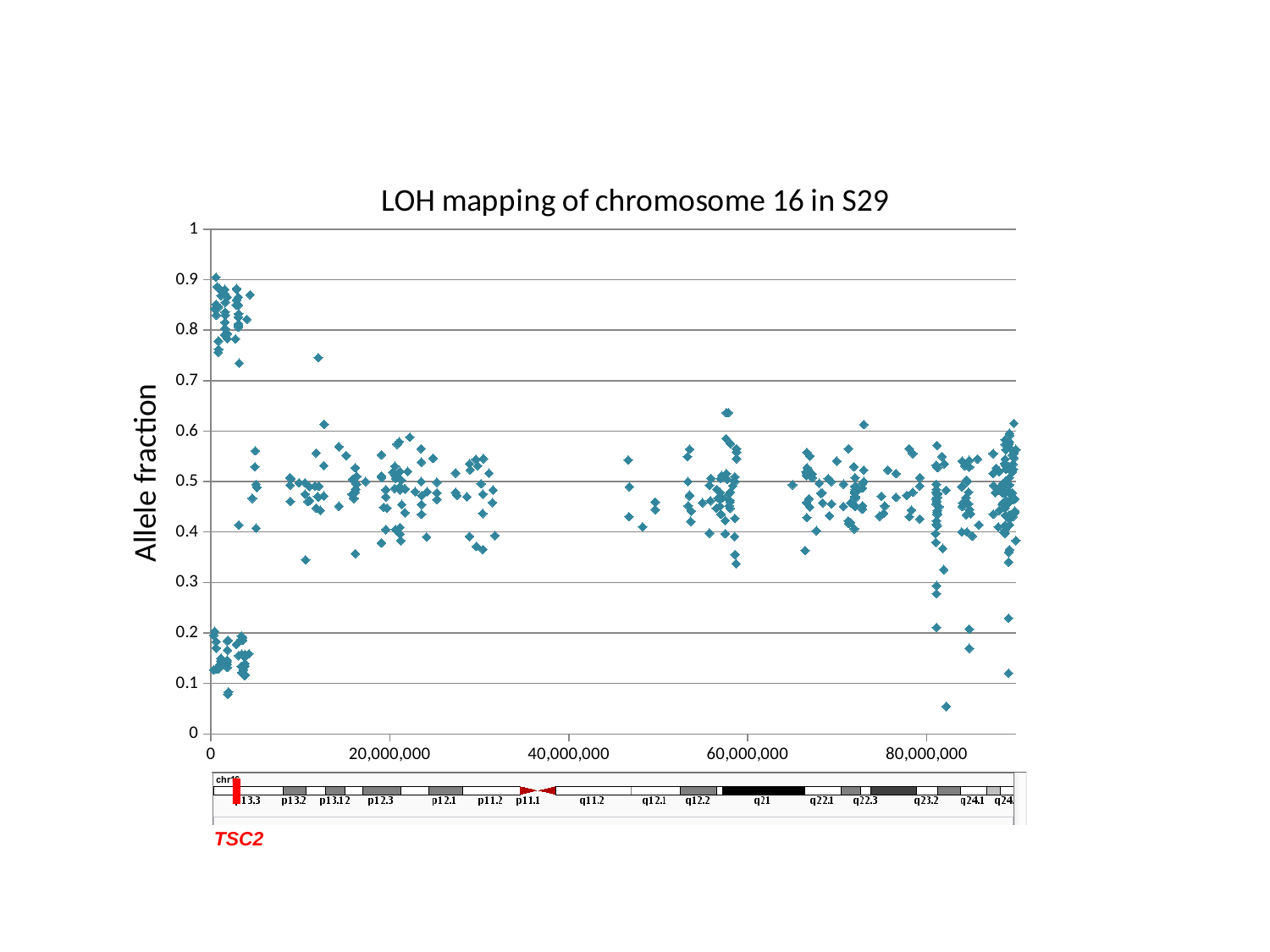

### Chart: LOH mapping of chromosome 16 in S29
| Category | t_ratio |
|---|---|Allele fraction
TSC2

## Slide 30
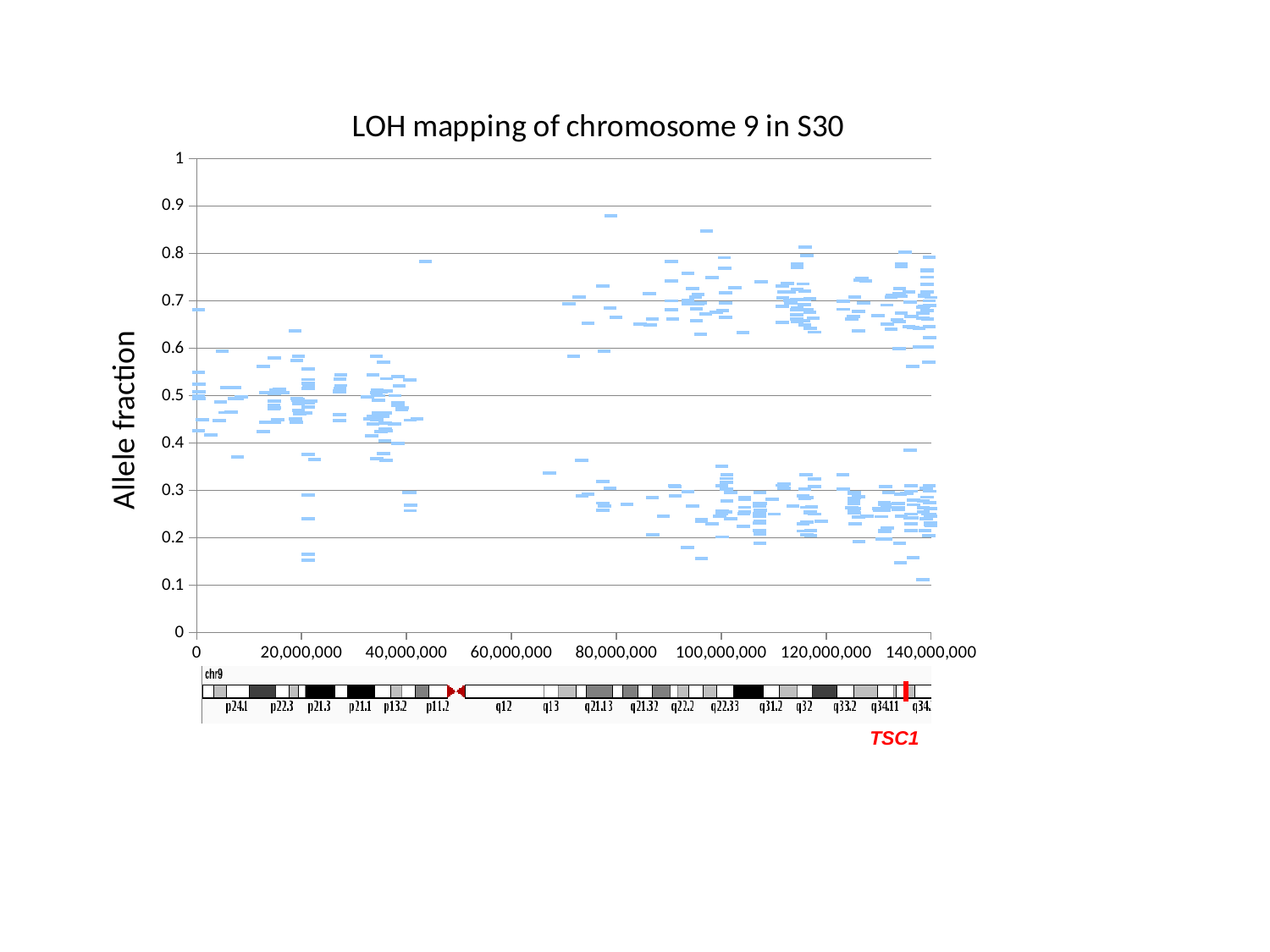

### Chart: LOH mapping of chromosome 9 in S30
| Category | t_ratio |
|---|---|Allele fraction
TSC1

## Slide 31
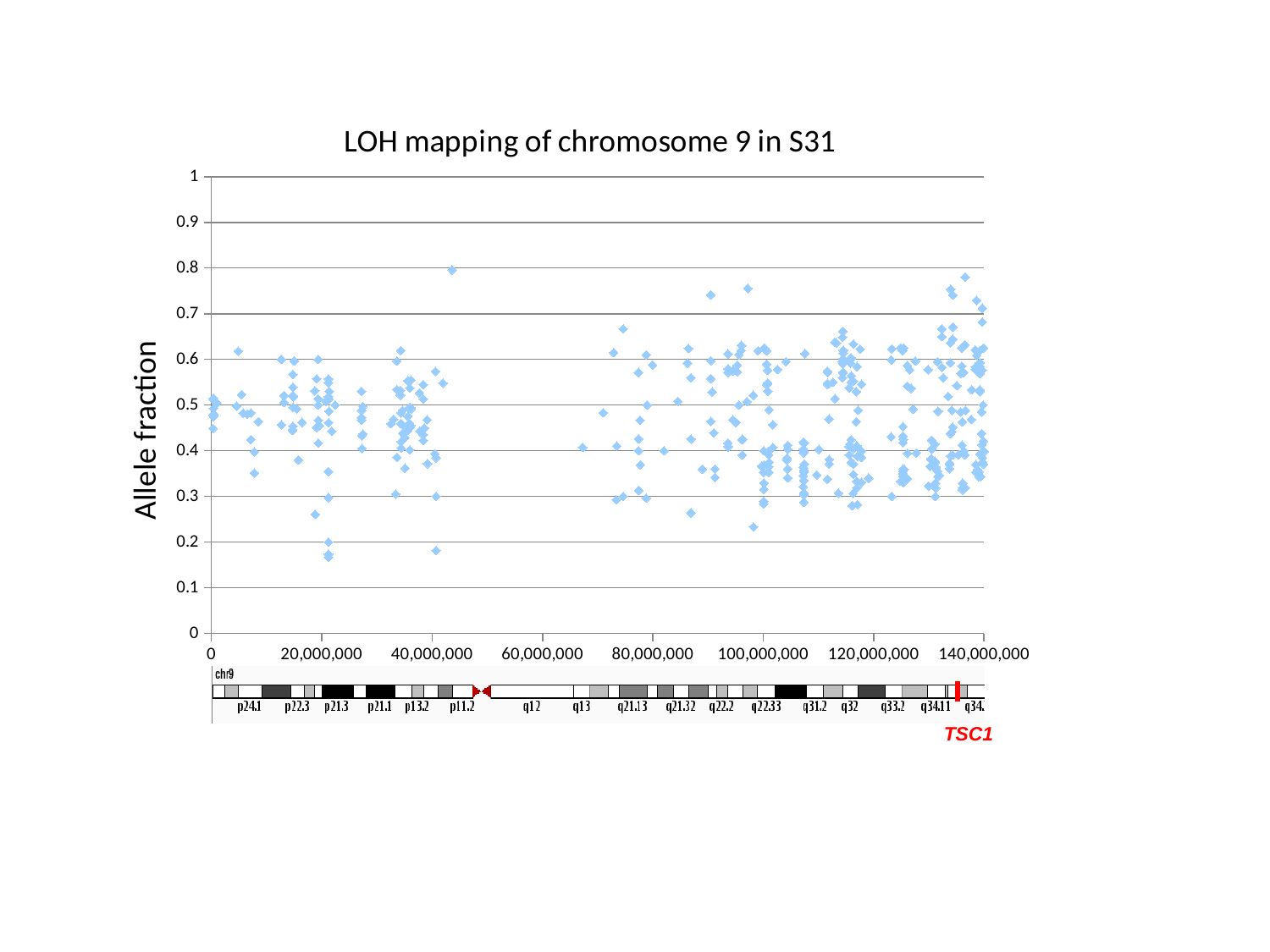

### Chart: LOH mapping of chromosome 9 in S31
| Category | t_ratio |
|---|---|Allele fraction
TSC1

## Slide 32
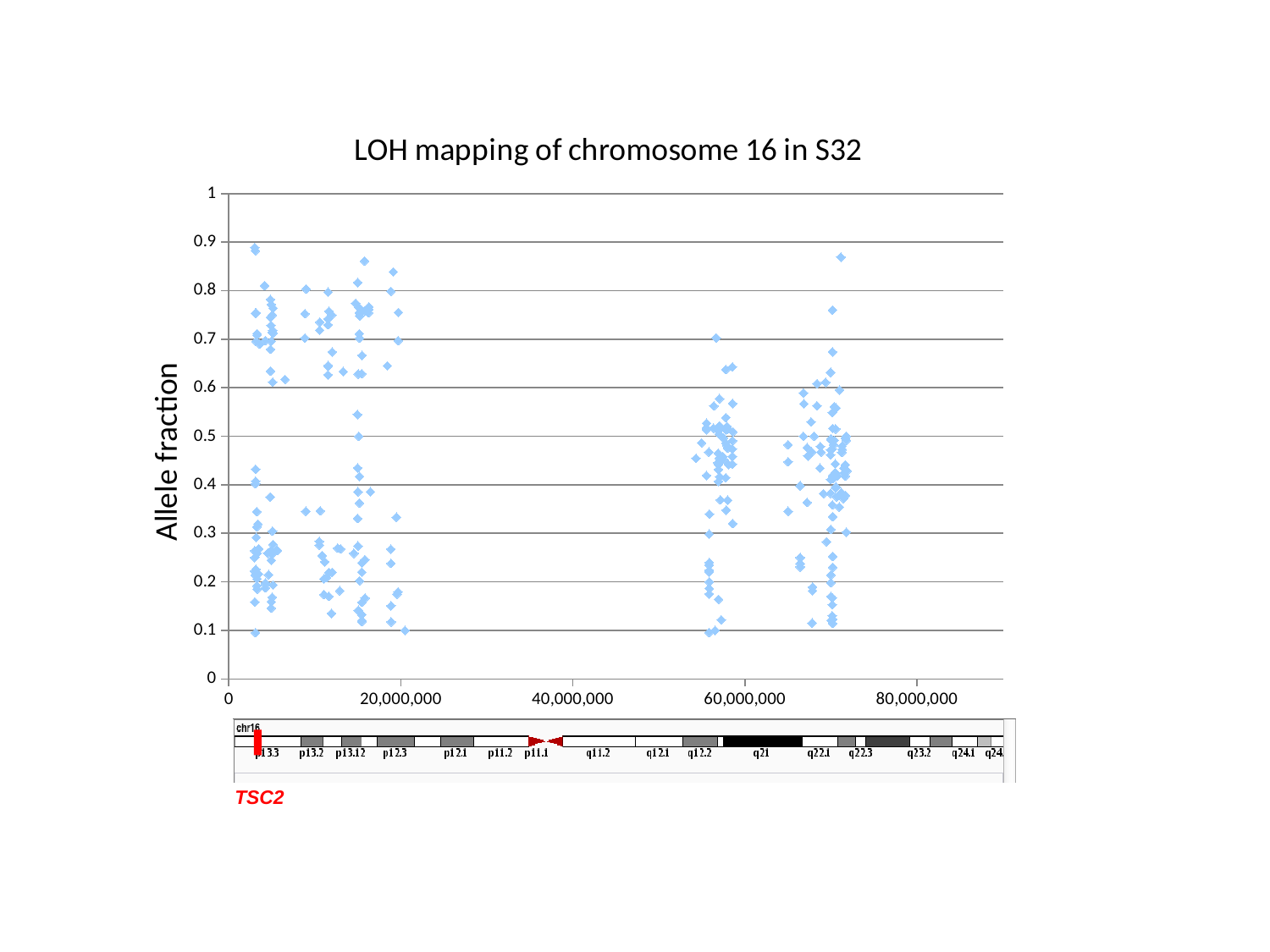

### Chart: LOH mapping of chromosome 16 in S32
| Category | t_ratio |
|---|---|Allele fraction
TSC2
