# Supplementary material for: Whole Exome Sequencing Identifies TSC1/TSC2 Biallelic Loss as the Primary and Sufficient Driver Event for Renal Angiomyolipoma Development
Source: PLoS Genet. 2016 Aug 5;12(8):e1006242. doi: 10.1371/journal.pgen.1006242 (PMC4975391; doi:10.1371/journal.pgen.1006242)
Supplement: S1 Table — (DOCX) [file pgen.1006242.s004.docx]

Glossary of genetic terms used in this publication:

Germ-line mutation. This is a mutation that is present in all of the cells of an individual, including the germ cell lineage (oocytes and sperm) and is transmittable to offspring.

Mosaicism. An individual who is mosaic is composed of two or more cell lines of different genetic or chromosomal constitution, both cell lines being derived from the same zygote (fertilized egg). Since mutations accumulate in all cells in all individuals during the course of development, we are all mosaics. A mosaic mutation occurs in a single cell at some stage during fetal development, and is then inherited by all of the progeny cells of that cell. The fraction of cells that contain a mosaic mutation can range from very low (< 0.1%) to relatively high (e.g. 50%). In addition, the fraction of cells with a mosaic mutation can vary among different tissue and cell types. When mosaicism is present in many cell types it is called somatic mosaicism. When mosaicism is confined to the germ cells of the individuals, it is called germ-line mosaicism.

Somatic mutation. Any mutation that occurs in a cell that is not nor will become a germ cell is considered a somatic mutation. The term is commonly used to distinguish mutations that occur in tumor cells only, and not in other cells in the body.

Loss of Heterozygosity. Germ line genetic variants, e.g. single nucleotide polymorphisms (SNPs), consist of two alleles (e.g. A and G) that are seen at equal representation or 50%-50% allele frequency in normal tissue samples. In tumors, there can be loss of one allele of a SNP through large genomic deletion including a chromosome arm or entire chromosome. Loss of a chromosome region can be identified by examining SNP allele frequency data, and observing that it is significantly skewed from a 50:50 allele ratio. In a pure tumor cell sample, the allele ratio would be shifted from 50:50 to 100:0, with complete loss of one allele. In angiomyolipoma and most other cancer types, there is admixture of normal cells in the tumor sample, such that the allele ratio might be 75:25 for the two alleles. This alteration in allele ratio is called loss of heterozygosity (LOH).

Copy neutral LOH (CN LOH). CN LOH is a genetic event in which a chromosomal region of one homologous chromosome is replaced by the same chromosomal region from the other chromosome with no change in the number of copies (copy neutral). This is thought to occur through a process of mitotic recombination, in which there is exchange of genetic material from one chromosome to another. In the case of *TSC2* presented here (Figure 3, S3 Figure), we found that although there was clear evidence of LOH for markers near *TSC2* on chromosome 16, the total number of copies of all segments of chromosome 16 was normal (diploid). The results indicate that a portion of chromosome 16p containing the mutant *TSC2* allele was transferred to the other chromosome 16. This leads to LOH observed over a region of chromosome 16p, including the *TSC2* mutation present on that chromosome.
